# Loss of *O*-Linked Protein Glycosylation in Burkholderia cenocepacia Impairs Biofilm Formation and Siderophore Activity and Alters Transcriptional Regulators

**DOI:** 10.1128/mSphere.00660-19

**Published:** 2019-11-13

**Authors:** Cameron C. Oppy, Leila Jebeli, Miku Kuba, Clare V. Oates, Richard Strugnell, Laura E. Edgington-Mitchell, Miguel A. Valvano, Elizabeth L. Hartland, Hayley J. Newton, Nichollas E. Scott

**Affiliations:** a Department of Microbiology and Immunology, University of Melbourne at the Peter Doherty Institute for Infection and Immunity, Melbourne, Australia; b Bio21 Molecular Science and Biotechnology Institute, University of Melbourne, Victoria, Australia; c Drug Discovery Biology, Monash Institute of Pharmaceutical Sciences, Monash University, Parkville, Victoria, Australia; d Department of Oral and Maxillofacial Surgery, New York University College of Dentistry, Bluestone Center for Clinical Research, New York, New York, USA; e Wellcome-Wolfson Institute for Experimental Medicine, Queen’s University Belfast, Belfast, United Kingdom; f Department of Microbiology and Immunology, University of Western Ontario, London, Ontario, Canada; g Centre for Innate Immunity and Infectious Diseases, Hudson Institute of Medical Research, Clayton, Victoria, Australia; h Department of Molecular and Translational Science, Monash University, Clayton, Victoria, Australia; University of Rochester

**Keywords:** glycosylation, pathogenesis, *Burkholderia cenocepacia*, posttranslational modifications, proteomics, DNA binding, CepR, glycoproteins, protein modification

## Abstract

*O*-linked protein glycosylation is a conserved feature of the *Burkholderia* genus. The addition of the trisaccharide β-Gal-(1,3)-α-GalNAc-(1,3)-β-GalNAc to membrane exported proteins in Burkholderia cenocepacia is required for bacterial fitness and resistance to environmental stress. However, the underlying causes of the defects observed in the absence of glycosylation are unclear. Using proteomics, luciferase reporter assays, and DNA cross-linking, we demonstrate the loss of glycosylation leads to changes in transcriptional regulation of multiple proteins, including the repression of the master quorum CepR/I. These proteomic and transcriptional alterations lead to the abolition of biofilm formation and defects in siderophore activity. Surprisingly, the abundance of most of the known glycosylated proteins did not significantly change in the glycosylation-defective mutants, except for BCAL1086 and BCAL2974, which were found in reduced amounts, suggesting they could be degraded. However, the loss of these two proteins was not responsible for driving the proteomic alterations, biofilm formation, or siderophore activity. Together, our results show that loss of glycosylation in B. cenocepacia results in a global cell reprogramming via alteration of the transcriptional regulatory systems, which cannot be explained by the abundance changes in known B. cenocepacia glycoproteins.

**IMPORTANCE** Protein glycosylation is increasingly recognized as a common posttranslational protein modification in bacterial species. Despite this commonality, our understanding of the role of most glycosylation systems in bacterial physiology and pathogenesis is incomplete. In this work, we investigated the effect of the disruption of *O*-linked glycosylation in the opportunistic pathogen Burkholderia cenocepacia using a combination of proteomic, molecular, and phenotypic assays. We find that in contrast to recent findings on the *N*-linked glycosylation systems of Campylobacter jejuni, *O*-linked glycosylation does not appear to play a role in proteome stabilization of most glycoproteins. Our results reveal that loss of glycosylation in B. cenocepacia strains leads to global proteome and transcriptional changes, including the repression of the quorum-sensing regulator *cepR* (*BCAM1868*) gene. These alterations lead to dramatic phenotypic changes in glycosylation-null strains, which are paralleled by both global proteomic and transcriptional alterations, which do not appear to directly result from the loss of glycosylation per se. This research unravels the pleiotropic effects of *O*-linked glycosylation in B. cenocepacia, demonstrating that its loss does not simply affect the stability of the glycoproteome, but also interferes with transcription and the broader proteome.

## Abstract (precis)

Protein glycosylation is increasingly recognized as a common posttranslational protein modification in bacterial species. Despite this commonality, our understanding of the role of most glycosylation systems in bacterial physiology and pathogenesis is incomplete. In this work, we investigated the effect of the disruption of *O*-linked glycosylation in the opportunistic pathogen Burkholderia cenocepacia using a combination of proteomic, molecular, and phenotypic assays. We find that in contrast to recent findings on the *N*-linked glycosylation systems of Campylobacter jejuni, *O*-linked glycosylation does not appear to play a role in proteome stabilization of most glycoproteins. Our results reveal that loss of glycosylation in B. cenocepacia strains leads to global proteome and transcriptional changes, including the repression of the quorum-sensing regulator *cepR* (*BCAM1868*) gene. These alterations lead to dramatic phenotypic changes in glycosylation-null strains, which are paralleled by both global proteomic and transcriptional alterations, which do not appear to directly result from the loss of glycosylation per se. This research unravels the pleiotropic effects of *O*-linked glycosylation in B. cenocepacia, demonstrating that its loss does not simply affect the stability of the glycoproteome, but also interferes with transcription and the broader proteome.

## INTRODUCTION

The Burkholderia cepacia complex (Bcc) includes diverse and ubiquitous, phylogenetically related Gram-negative species (1). To date, 20 Bcc species have been identified (1–3), but the commonality of Bcc in the environment (2, 3) and their recognition as opportunistic pathogens (4–6) continually drives the identification of new Bcc members. Within clinical settings, Bcc can lead to fatal infections (7, 8) that are challenging to control with antibiotic therapies (9) and can be spread by patient-to-patient transmission (10, 11). This is especially problematic for Bcc infections in people with cystic fibrosis (CF), where Bcc infections result in accelerated loss of lung function (12) as well as increased morbidity and mortality compared to other infectious agents (13, 14). B. cenocepacia is one of the most common Bcc species isolated from CF patients across the globe (15–18) and is generally associated with more fulminant disease leading to higher mortality than observed with other Bcc species (19). One of the most serious clinical outcomes from B. cenocepacia infections in people with CF is a condition known as “cepacia syndrome,” an unrelenting necrotizing pneumonia that rapidly leads to respiratory failure, bacteremia, and death (20). Although interventions with antimicrobial therapies can stop or even reverse cepacia syndrome (20), the intrinsic resistance of Bcc to multiple classes of antibiotic (21–23) and their propensity to form biofilms (24) make treatment success variable at best (9). To improve clinical outcomes, it is therefore essential to better understand the factors contributing to the ability of B. cenocepacia to infect immunocompromised hosts.

Biofilm formation is associated with bacterial persistence and the failure of antimicrobial treatments in a range of pathogens (25). Bcc members, including B. cenocepacia, produce biofilms on abiotic (26, 27) and biotic (28) surfaces. However, B. cenocepacia bacteria in the CF lung do not appear to form true biofilms, but instead are observed extracellularly as small clusters surrounded by mucus and mainly within phagocytic cells in the submucosal tissue (29, 30). Increased biofilm production is associated with bacterial persistence in CF patients (31), and mutations selected for during chronic infections in CF patients mirror those observed during biofilm *in vitro* evolution experiments (32). The ability to form biofilms in Bcc, as well as the expression of multiple virulence factors, is controlled by numerous quorum sensing (QS) systems (33). A key class of QS systems associated with Bcc virulence are based on homoserine lactones (HSLs) (24). Across the Bcc, some HSL QS systems are variable or lineage specific, such as CciR/I and CepR2 (34, 35), while others are highly conserved in all members. One such highly conserved HSL QS system is the CepR/I regulon (36, 37), which generates *N*-octanoylhomoserine lactone (C_8_-HSL) using the HSL synthase CepI (BCAM1870), which in turns activates the transcriptional regulator CepR (37, 38). CepR (BCAM1868) is a major regulator of biofilm formation (39), and disruption of CepR/I attenuates Bcc virulence in several models (40, 41) and reduces disease severity (40, 42). The importance of the CepR/I QS system in Bcc virulence stems from its broad regulatory profile affecting multiple virulence-associated genes (43–45), such as those encoding the secreted zinc metalloproteases ZmpA (46) and ZmpB (47), siderophore production (39, 48), and the key mediator of biofilm formation protein A (BapA) (45).

Glycosylation is increasingly recognized as a common posttranslational modification in bacterial systems (49–56). Many glycosylation systems are conserved across bacterial genera (57, 58) and phyla (59, 60), suggesting glycosylation is critical for optimal proteome functionality. Disruption of glycosylation pathways in several species results in reduced fitness compared to glycosylation-competent strains (52–56). However, the underlying cause of fitness reduction remains poorly defined (61, 62). Only recently have mechanistic insights emerged on how the loss of glycosylation affects bacterial physiology and pathogenesis. In Campylobacter jejuni, loss of glycosylation results in decreased stability of the majority of known glycoproteins, which in turn affects virulence (63, 64). These data support a model whereby bacterial *N*-linked glycosylation contributes to protein stability, but it is unclear whether other glycosylation systems, such as *O*-linked glycosylation, have evolved to stabilize glycosylated proteins.

Previously, we reported B. cenocepacia possesses an *O*-linked glycosylation system responsible for the modification of at least 23 proteins with a trisaccharide glycan using the enzyme PglL (BCAL0960) (56). Building on this work, we recently identified the biosynthetic locus, the *O*-glycosylation cluster (OGC [BCAL3114 to BCAL3118]), responsible for the generation of the *O*-linked glycan, established the *O*-linked glycan structure as β-Gal-(1,3)-α-GalNAc-(1,3)-β-GalNAc, and demonstrated that glycosylation was required for optimal bacterial fitness and resistance to clearance in the Galleria mellonella infection models (65). Although these studies have demonstrated a link between glycosylation and bacterial fitness, the mechanism remains unclear. Using quantitative proteomic approaches, we sought to understand the proteome changes resulting from the loss of *O*-linked glycosylation in B. cenocepacia. We demonstrated that loss of glycosylation in B. cenocepacia resulted in global proteome alterations beyond the known glycoproteome, which are associated with widespread alterations in transcriptional regulation. We discovered that the HSL QS system CepR/I is repressed in glycosylation-defective mutants, and this coincides with defective biofilm formation and reduced siderophore activity. In contrast to the loss of glycosylation in C. jejuni, we also demonstrate that only a few glycoproteins are reduced in abundance in the absence of glycosylation, but they are not responsible for the glycosylation-null phenotypes. Together, our data indicate that the roles of glycosylation in B. cenocepacia extend beyond protein stabilization, and loss of *O*-linked glycosylation in B. cenocepacia causes dramatic physiological changes due to alterations in transcriptional regulatory systems and the proteome at large.

## RESULTS

### Loss of glycosylation in B. cenocepacia leads to global proteome alterations.

**FIG 1:**
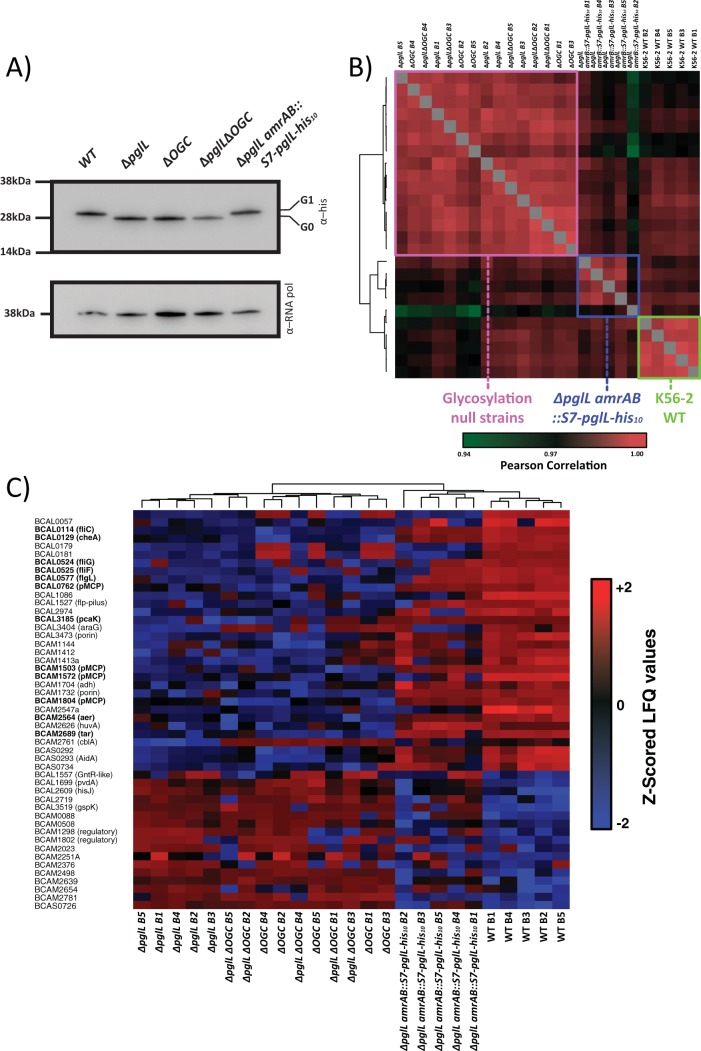
Disruption of *O*-linked glycosylation results in multiple changes in the proteome. (A) Western analysis of strains expressing the glycosylation substrate DsbA_Nm_-His_6_ confirms the loss of glycosylation in the *ΔpglL*, *Δ*OGC, and *ΔpglL Δ*OGC mutant strains and restoration of glycosylation in the *ΔpglL amrAB*::S7*-pglL-*His_10_ chromosomal complemented strain. (B) Pearson correlation analysis demonstrates three discrete clusters observed across the proteomic analysis which separate glycosylation-competent and glycosylation-null strains. (C) Z-scored heat map of proteins observed to undergo alterations between glycosylation-competent and glycosylation-null strains reveals alterations in motility and chemotaxis (proteins in boldface), including BCAL0114 (FliC), BCAL0524 (FliG), and BCAL0525 (FliF), as well as known CepR-regulated protein BCAS0293 (AidA).

We previously demonstrated that loss of glycosylation causes defects in motility (56), reduction of virulence in plant and insect infection models (56, 65), and defects in carbon utilization (65). To better understand the role of glycosylation in B. cenocepacia, we assessed the effect of loss of glycosylation on the proteome. To achieve this, we generated markerless deletion mutations in the *O*-oligosaccharyltransferase *pglL* gene (*ΔpglL* [*BCAL0960*]) (56), the recently identified *O*-linked glycan cluster (*Δ*OGC [*BCAL3114* to *BCAL3118*]) responsible for the generation of the glycan used for *O*-linked glycosylation (65), and a double-glycosylation-null strain (*ΔpglL Δ*OGC). We also constructed a chromosomal *pglL* complemented strain (*ΔpglL amrAB*::S7*-pglL-*His_10_) (see Fig. S1A in the supplemental material). The rationale for creating multiple glycosylation-defective strains was to eliminate potential confounding effects arising from blocking glycosylation at a specific step and the corresponding accumulation of unprocessed lipid-linked glycans. Western blot analysis using the glycoprotein acceptor protein DsbA_Nm_-His_6_ (56, 66) supported the loss of glycosylation in the *ΔpglL*, *Δ*OGC, and *ΔpglL Δ*OGC strains, as well as restoration of glycosylation in the *ΔpglL amrAB*::S7*-pglL-*His_10_ strain (Fig. 1A). In contrast to our previously reported plasmid-based PglL complementation approaches (56) chromosomal complementation lead to the restoration of glycosylation to near wild-type (WT) levels (Fig. S1B) as well as restoration of motility (Fig. S1C) compared to only partial restoration previously reported (56).

10.1128/mSphere.00660-19.2 FIG S1 Confirmation of glycosylation status and motility in *Burkholderia* strains. (A) Western blots confirm the expression of *pglL-*His_10_ in the *amrAB*::S7*-pglL-*His_10_ complement strain. (B) Whole-cell liquid chromatography-tandem mass spectrometry (LC-MS/MS) proteomic analysis confirms the presence of glycopeptides in WT and *amrAB*::S7*-pglL-*His_10_ strain lysates at comparable levels which are absent in the Δ*pglL* mutant. Conversely, unmodified forms of multiple glycopeptides are also observed in the Δ*pglL* mutant that are absent from the WT and *amrAB*::S7*-pglL-*His_10_ strains. (C) Consistent with the restoration of glycosylation in the *amrAB*::S7*-pglL-*His_10_ strain is the restoration of motility to near-wild-type levels. (D) Monitoring of endogenous glycopeptide in the whole-cell proteome samples confirms loss of glycosylation in the Δ*pglL*, ΔOGC Δ*pglL*, and ΔOGC mutants by the absence of glycopeptides from the glycoproteins B4EB73, B4EET7, and B4EBR7. Download FIG S1, TIF file, 1.2 MB. Copyright © 2019 Oppy et al. 2019 Oppy et al. This content is distributed under the terms of the Creative Commons Attribution 4.0 International license.

Using label-free quantification (LFQ)-based quantitative proteomics, 5 biological replicates of each strain were investigated, leading to the identification of 3,399 proteins with 2,759 proteins quantified in at least 3 biological replicates in a single biological group (see Fig. S2A and B and Data Set S1, tab 1, in the supplemental material). As expected, no glycopeptides were observed in the *ΔpglL*, *ΔpglL Δ*OGC, and *Δ*OGC strains, while multiple glycopeptides were observed in the wild-type and *ΔpglL amrAB*::S7*-pglL-*His_10_ strains (Fig. S1D). Hierarchical clustering of Pearson correlations of proteome samples demonstrated robust correlation between all samples (average Pearson correlation of 0.98 [Data Set S1, tab 2]); yet three discrete proteome clusters were readily identified separating the wild-type K56-2 and *ΔpglL amrAB*::S7*-pglL-*His_10_ strains and the glycosylation-null strains (Fig. 1B). Examination of the most profound alterations, proteins with a –log_10_ *P* value of >3 and a fold change greater than ±2 log_2_ units, revealed alterations in protein levels observed in the *ΔpglL* mutant that were mirrored in the *Δ*OGC and *ΔpglL Δ*OGC strains, which were restored by complementation (Fig. 1C). Consistent with the observed motility defects (Fig. S1C), the levels of proteins associated with flagellum-mediated motility and chemotaxis, including BCAL0114 (FliC), BCAL0129 (CheA), BCAL0524 (FliG), and BCAL0525 (FliF), were significantly reduced in glycosylation-null strains. Importantly, multiple known virulence-associated proteins were also decreased in the glycosylation-null strains, including the heme receptor protein HuvA (BCAM2626 [67]) and nematocidal protein AidA (BCAS0293 [68]). Numeration of the overlap of all altered protein between glycosylation-null strains by Fisher exact enrichment analysis demonstrated a substantial enrichment between these three groups (Fisher’s exact test, 6.7502 × 10^177^ and 4.3784 × 10^245^ for the *ΔpglL* compared with *Δ*OGC strain, and for the *ΔpglL* compared with *ΔpglL Δ*OGC strain, respectively) (Data Set S1, tab 3, and Fig. S2C). These results revealed that the loss of glycosylation due to disruption of *pglL* or OGC leads to similar changes, which are largely complemented to parental levels by reintroduction of *pglL* in the chromosome.

10.1128/mSphere.00660-19.3 FIG S2 Proteome coverage and overlap in altered proteins between biological groups. (A) Across replicates, the majority of proteins are observed in >20 replicates, allowing robust analysis of proteome changes. (B) In at least one biological group, 2,759 proteins are quantified across three biological replicates. (C) The overlap of altered proteins in the Δ*pglL*, Δ*pglL* ΔOGC, and ΔOGC mutants demonstrate the majority of proteins are altered in all glycosylation mutants compared to the WT. Download FIG S2, EPS file, 1.2 MB. Copyright © 2019 Oppy et al. 2019 Oppy et al. This content is distributed under the terms of the Creative Commons Attribution 4.0 International license.

10.1128/mSphere.00660-19.9 DATA SET S1 Proteomic analysis of the B. cenocepacia strains. All proteomic analyses associated with quantifying the impact of glycosylation on the proteome of B. cenocepacia are provided. Download Data Set S1, XLSX file, 6.4 MB. Copyright © 2019 Oppy et al. 2019 Oppy et al. This content is distributed under the terms of the Creative Commons Attribution 4.0 International license.

### Loss of glycosylation results in reduction in CepR/I transcription and the levels of DNA-associated CepR.

**FIG 2:**
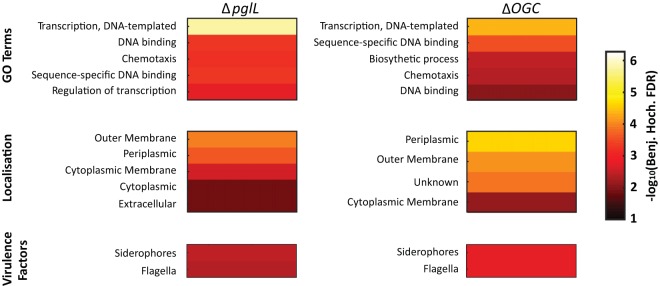
Heat maps of glycosylation-null strain enrichment analysis. The multiple hypothesis corrected *P* values from Fisher’s exact tests demonstrate that proteins with similar GO terms and localizations and associated with virulence factors are altered in glycosylation-null strains.

Enrichment analysis of the altered proteins in glycosylation-null strains demonstrate the over representation of a range of categorical groups based on GO (Gene Ontology) terms, protein localization, and virulence-associated factor assignments. These groups highlight that protein localization assignments and virulence-associated factors were similarly affected in *ΔpglL* and *Δ*OGC strains, recapitulating observations made at the individual protein level (Fig. 2; Data Set S1, tab 3). Interestingly, enrichment analysis highlighted the link between the loss of *O*-linked glycosylation and changes that were broader than only motility and virulence. For example, differences also observed in proteins associated with DNA-sequence specific binding and transcriptional regulation (Fig. 2; Data Set S1, tab 3). This observation suggested that loss of glycosylation results in alterations in the transcriptional landscape of B. cenocepacia. As virulence is coordinated by global regulators such as CciR, CepR, ShvR, and AtsR in B. cenocepacia (35, 43, 69, 70), we assessed if known regulators could account for the observed proteome changes in glycosylation-null strains. As our data demonstrated minimal alteration of the regulator ShvR (BCAS0225; Data Set S1, tab 1) across the analyzed strains, and disruption of both *atsR* (*BCAM0379*) and *cciR* (*BCAM0240*) has previously been associated with increased motility (43, 69), we reasoned that the regulator CepR (BCAM1868) may be responsible for the glycosylation-dependent differences in our mutant strains. Although CepR is observed within our proteomic analysis, its low intensity prevented accurate quantitation across all strains (Data Set S1, tab 1). However, the stringently CepR-regulated AidA protein (BCAS0293 [45, 71]) exhibited decreases of −2.9 and −3.1 log_2_ within *ΔpglL* and *Δ*OGC strains compared to the WT (Fig. 1C), indicating reduced CepR levels. This observation prompted us to investigate regulation of other known CepR-regulated genes and proteins. Using available microarray data of CepR-regulated genes (43), we investigated the correlation of the proteome changes observed in the absence of glycosylation, with alterations observed in response to the disruption of CepR. We observed a statistically significant enrichment of CepR-regulated proteins altered in the absence of glycosylation (multiple hypothesis corrected *P* values of 1.79 × 10^6^ and 6.69 × 10^6^ for the *ΔpglL* and *Δ*OGC strains, respectively [Data Set S1, tab 3]), supporting a link between CepR and the alteration observed in glycosylation-null strains and suggesting that the loss of glycosylation may influence the B. cenocepacia CepR regulon.

**FIG 3:**
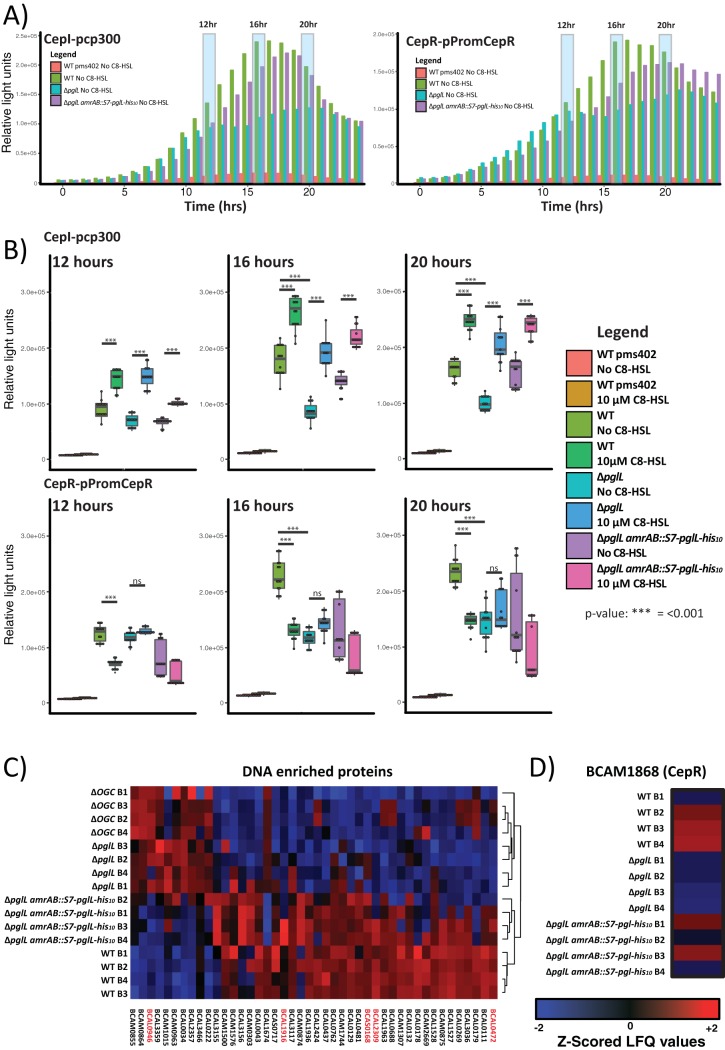
CepR/I transcription is altered in glycosylation-null strains. (A) Twenty-four-hour luciferase profile of strains grown with either the CepI reporter pCP300 or CepR reporter pPromCepR demonstrating alteration in luciferase activity in the Δ*pglL* mutant compared to the WT and Δ*pglL amrAB*::S7*-pglL-*His_10_ complemented strains. Each data point corresponds to the mean of three independent biological replicates with a more detailed figure containing the plotted standard deviation provided in Fig. S3. (B) Detailed analysis of three time points across the luciferase profiles are provided for the 12-h (log phase), 16-h (transition from log to stationary phase), and 20-h (stationary phase) time points. For each time point, the luciferase activities of strains grown with and without C_8_-HSL are shown. (C) Z-scored heat map of DNA-bound proteins with significant alterations in abundance in the Δ*pglL* or ΔOGC mutant compared to the WT reveal similar protein profiles for glycosylation-null strains compared to glycosylation-competent strains. (D) DNA bound proteome analysis of CepR supports the reduction in the abundance of DNA-bound CepR in the Δ*pglL* strain and the partial restoration of CepR in the Δ*pglL amrAB*::S7*-pglL-*His_10_ strain.

To determine transcriptional changes in *cepR/I* genes, we introduced the *cepR* and *cepI* luciferase promoter reporter (pPromcepR [69] and pCP300 [72]) into the wild-type K56-2, mutant *ΔpglL*, and complemented *ΔpglL amrAB*::S7*-pglL-*His_10_ strains. As expected from the proteomic results, the *ΔpglL* strain showed decreased induction of both *cepI* and *cepR* over a 24-h period (Fig. 3A; see Fig. S3 in the supplemental material) compared with the wild-type and *ΔpglL amrAB*::S7*-pglL-*His_10_ strains. Detailed examination at 12 h (log phase), 16 h (the transition from log to stationary phase), and 20 h (stationary phase) revealed higher levels of transcription in the wild type of both *cepI* and *cepR* at 16 and 20 h compared with transcription levels in the *ΔpglL* mutant, despite comparable growth kinetics (see Fig. S4A and B in the supplemental material). As the C_8_-HSL levels affect the response of CepI and CepR in B. cenocepacia (39, 44, 73), we assayed *cepR*/*I* transcription in the absence and presence of additional C_8_-HSL (10 μM [Fig. 3B]). In response to exogenous C_8_-HSL, *cepI* transcription increased in all strains (Fig. 3B), consistent with the positive-feedback response expected to heighten C_8_-HSL levels (39, 44). In contrast, while the addition of C_8_-HSL led to no change in *cepR* transcription in the *ΔpglL* mutant, it resulted in reduced transcription of *cepR* to the level observed in the wild-type K56-2 strain. Complementation of *pglL*, using *amrAB*::S7*-pglL-*His_10_, restored CepI transcription to wild-type levels but only partially restored CepR transcription (Fig. 3B). As expected from the reduction in *cepR*/*I* transcription resulting from the loss of glycosylation, *cepR* and *cepI* transcription was also compromised in *Δ*OGC strains (Fig. S4C to F). Together, these results indicate that both *cepR* and *cepI* transcription are altered in the loss of glycosylation, with the resulting *cepR* levels resembling the levels observed during C_8_-HSL-induced repression in wild type.

10.1128/mSphere.00660-19.4 FIG S3 Luciferase measure of WT versus the Δ*pglL* mutant and the WT versus Δ*pglL amrAB*::S7*-pglL-*His_10_ strain containing the luciferase promoter reporters PromCepR (CepR reporter [A and B]) or pcp300 (CepI reporter [C and D]). Twenty-four-hour luciferase curves of strains containing the *lux* reporter in LB plus trimethoprim (TMP [100 μg/ml]) demonstrates a reduction in the induction of both CepR and CepI in Δ*pglL* strains, which is partially restored upon complementation. Standard derivation of biological replicates is denoted by shading. As a control, the promoterless *lux* reporter plasmid pMS402 in the K56-2 WT strain is also shown. Download FIG S3, TIF file, 1.6 MB. Copyright © 2019 Oppy et al. 2019 Oppy et al. This content is distributed under the terms of the Creative Commons Attribution 4.0 International license.

10.1128/mSphere.00660-19.5 FIG S4 Growth and Lux reporter curves of strains. (A and B) Twenty-four-hour curves of strains containing *lux* reporter in LB plus TMP (100 μg/ml) demonstrate comparable growth kinetics across strains for both the CepR promoter (pPromCepR) and CepI promoter (pcp300). The standard derivation of biological replicates is denoted by shading. (C) CepR promoter *lux* reporter measurements at 12, 16, and 20 h in the ΔOGC mutant demonstrate a decrease in the transcription of CepR upon entrance into stationary phase. (D) Twenty-four-hour curves of strains containing *lux* reporter in LB plus TMP (100 μg/ml) demonstrate comparable growth kinetics across strains. Standard derivation of biological replicates is denoted by shading. (E) CepI promoter *lux* reporter measurements at 12, 16, and 20 h in the ΔOGC mutant demonstrate a decrease in the transcription of CepI upon entrance into stationary phase. (F) Twenty-four-hour curves of strains containing the *lux* reporter in LB plus TMP (100 μg/ml) demonstrate comparable growth kinetics across strains. Standard derivation of biological replicates is denoted by shading. Download FIG S4, TIF file, 1.3 MB. Copyright © 2019 Oppy et al. 2019 Oppy et al. This content is distributed under the terms of the Creative Commons Attribution 4.0 International license.

As the CepR protein autoregulates *cepR*’s own transcription (48), we reasoned that the decreased transcription in the Δ*pglL* mutant would correspond to decreased levels of DNA-bound CepR. To directly assay DNA binding by CepR, we monitored the DNA-bound proteome using formaldehyde-based cross-linking coupled to DNA enrichment (74). Initial analysis of the DNA-bound proteome found glycosylation-null strains (*ΔpglL* and *Δ*OGC) and glycosylation-proficient strains (wild type and *ΔpglL armAB*::S7*-pglL-*His_10_) possessed distinct proteome profiles with multiple uncharacterized transcriptional regulators (e.g., BCAL0946, BCAL1916, BCAS0168, BCAL2309, and BCAL0472) which were altered by the loss of glycosylation (Fig. 3C; Data Set S1, tab 4). Although this analysis enabled the identification of CepR, its low abundance prevented its quantitation across biological replicates. To improve the monitoring of CepR, targeted proteomic analysis was undertaken using PRM assays, which confirmed the reduction in DNA-associated CepR in the *ΔpglL* mutant compared with the wild-type and *ΔpglL armAB*::S7*-pglL-*His_10_ strains (Fig. 3D [*P* = 0.017 for wild type versus Δ*pglL* strain]; Data Set S1, tab 5). In agreement with the total proteome and *lux* reporter measurements, the DNA-bound proteome supports multiple transcription-associated proteins, including the global regulator CepR, that are altered in the absence of glycosylation.

### The *ΔpglL* mutant demonstrates a reduced ability to form biofilms and produce siderophores.

**FIG 4:**
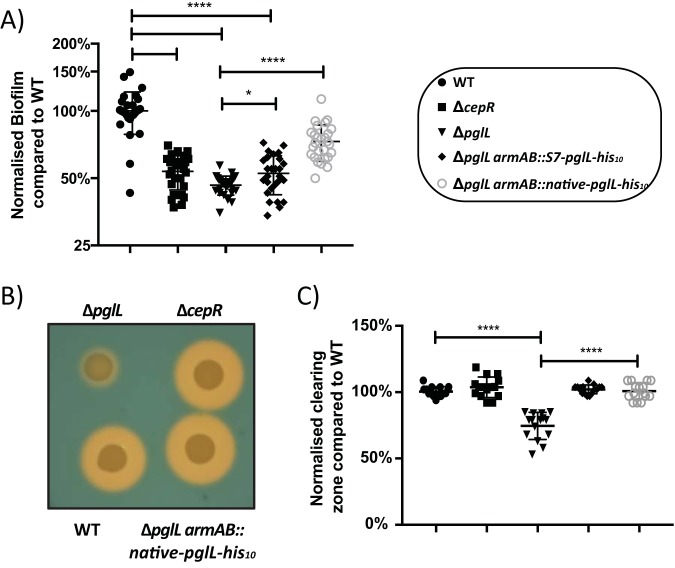
Biofilm formation and siderophore activities are reduced in the Δ*pglL* mutant. (A) Twenty-four-hour static biofilm assays demonstrate a decrease in biofilm formation in the Δ*pglL* strain, which is partially restored upon complementation. (B and C) CAS assays demonstrate a reduction in the zone of clearing in the Δ*pglL* mutant, which is restored upon complementation.

The observed reductions in CepR/I transcription suggested that CepR/I-linked phenotypes may also be altered in glycosylation-null strains. To test this hypothesis, we assessed two phenotypes associated with CepR/I regulation: (i) the production of biofilm under static 24-h growth and (ii) siderophore activity (39, 43–45, 48). Consistent with an impact of glycosylation on known CepR/I-regulated phenotypes, we observed a marked reduction in biofilm formation in the Δ*pglL* mutant, which was partially restored by complementation (Fig. 4A). Interestingly, we also observed that the method of complementation—i.e., expression of PglL-His_10_ driven from the native *pglL* promoter (Δ*pglL amrAB*::native*-pglL-*His_10_) or from the constitutive S7 promoter (Δ*pglL amrAB*::S7*-pglL-*His_10_)—affected the restoration of biofilm formation (Fig. 4A). Examination of independently created Δ*pglL* and Δ*pglL amrAB*::native*-pglL-*His_10_ strains confirmed a link between biofilm formation through phenotype restoration by complementation (see Fig. S5A in the supplemental material). Chrome azurol S (CAS) assays, used to assess the global levels of siderophore activity, demonstrated a reproducible effect in the Δ*pglL* mutant, which was completely restored by complementation when PglL was expressed from either its native or the S7 promoter (Fig. 4B and C). The ΔOGC and ΔOGC Δ*pglL* strains also demonstrate biofilm and siderophore alterations compared to the wild type, although these alterations were not completely identical to those observed in the Δ*pglL* mutant (Fig. S5B to D). Together, we conclude that phenotypes associated with CepR/I regulation, including biofilm and siderophore activity, are affected by the loss of glycosylation.

10.1128/mSphere.00660-19.6 FIG S5 Assessment of biofilm formation and siderophore activity across strains. (A) Independently created Δ*pglL* mutants demonstrate identical loss of biofilm phenotype and can partially restored by chromosomal complementation. (B) Disruption of OGC results in a marked increase in biofilm formation. (C and D) Disruption of OGC results in a marked decrease in siderophore activity. Download FIG S5, TIF file, 1.1 MB. Copyright © 2019 Oppy et al. 2019 Oppy et al. This content is distributed under the terms of the Creative Commons Attribution 4.0 International license.

### Except for BCAL1086 and BCAL2974, proteins that are normally glycosylated remain stable in the absence of glycosylation.

**FIG 5:**
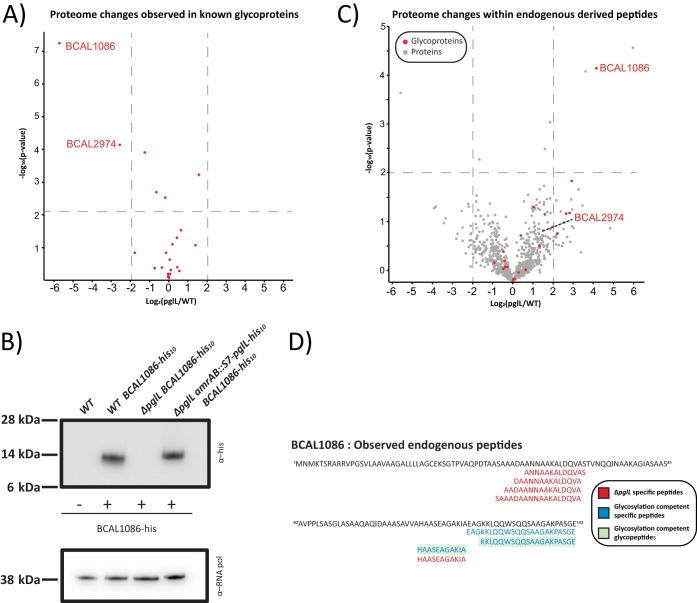
The stability of glycoproteins BCAL1086 and BCAL2974 is affected by loss of glycosylation. (A) Proteomic analysis demonstrates BCAL1086 and BCAL2974 decrease in abundance in the absence of glycosylation. (B) Endogenous tagging of BCAL1086 confirms the loss of BCAL1086 in the Δ*pglL* background. (C) Proteomic analysis of endogenous derived peptides demonstrates an increased abundance of BCAL1086-derived peptides in the absence of glycosylation. (D) Analysis of endogenous peptides confirms the presence of unique peptide fragments from BCAL1086 in the Δ*pglL* background.

As the loss of glycosylation in other bacterial glycosylation systems leads to protein instability (63, 64, 75), we examined whether protein instability in B. cenocepacia may be responsible for the phenotypic changes in glycosylation-null strains. Our proteomic analysis identified 21 out of 23 known glycoproteins (56), yet only 2 were altered in abundance in glycosylation-negative strains: BCAL1086 (−5.7 log_2_) and BCAL2974 (−2.5 log_2_) (Fig. 5A; Data Set S1, tab 1). To confirm the observed decreases in abundance, endogenous BCAL1086 and BCAL2974 were His_10_ tagged at the C terminus. While His tagging did not allow the detection of BCAL2974 by Western analysis (data not shown), the introduction of the His_10_ epitope into BCAL1086 allowed quantification of endogenous BCAL1086 in the K56-2 wild-type, Δ*pglL* mutant, and Δ*pglL amrAB*::S7*-pglL-*His_10_ complemented strain backgrounds and confirmed the loss of BCAL1086 in the Δ*pglL* mutant (Fig. 5B). We sought to directly assess whether BCAL1086 was subjected to increased degradation in the Δ*pglL* mutant, as a measure of instability. For this, we monitored the endogenous peptide pool (76), quantifying peptides derived from 783 proteins (Data Set S1, tabs 6 and 7) in the B. cenocepacia K56-2 wild-type, Δ*pglL* mutant, and Δ*pglL amrAB*::S7*-pglL-*His_10_ complemented strain. Consistent with the degradation of BCAL1086, we observed an increase in the abundance of BCAL1086-derived peptides in the *ΔpglL* mutant, while peptides from other known glycoproteins showed only modest changes (Fig. 5C; Data Set S1, tab 7). Within this peptidomic analysis, we observed that multiple unique BCAL1086 peptides were present in the *ΔpglL* mutant clustered around the central region of BCAL1086 (Fig. 5D), confirming that BCAL1086 was expressed in the *ΔpglL* mutant, but subjected to proteolysis. Together, our data support that BCAL1086 becomes degraded in the absence of glycosylation, but the majority of known B. cenocepacia glycoproteins are unaffected.

### Role of BCAL1086 and BCAL2974 in *ΔpglL* phenotypes.

**FIG 6:**
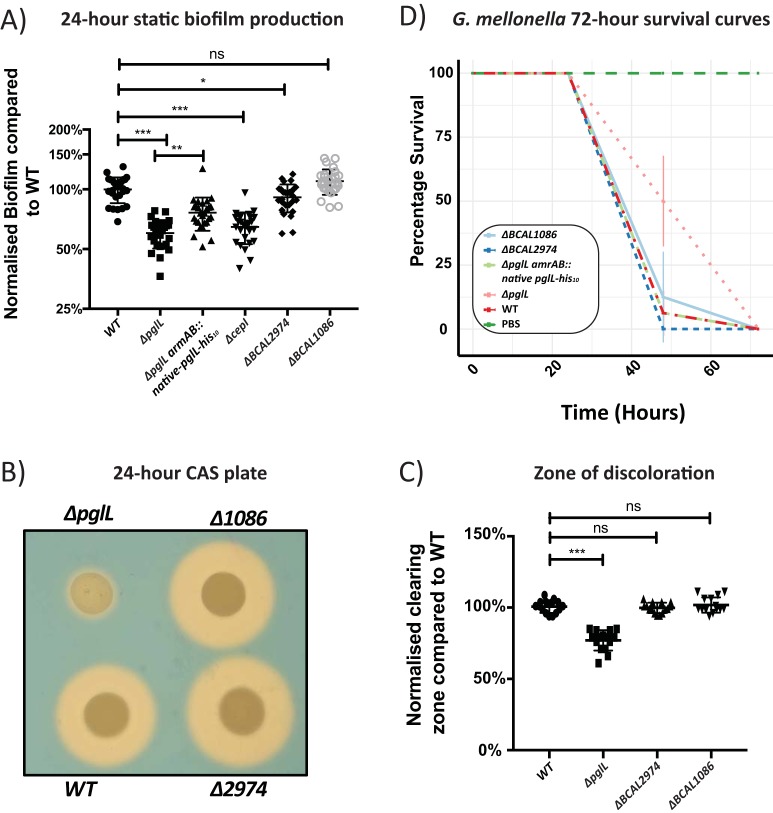
The loss of BCAL1086 or BCAL2974 does not affect phenotypes associated with Δ*pglL* mutation. (A) Twenty-four-hour static biofilm formation is unaffected in the Δ*BCAL1086* mutant and minimally affected in the Δ*BCAL2974* mutant compared to the WT. (B) CAS plate assays demonstrate similar zones of clearing in the Δ*BCAL1086* and Δ*BCAL2974* strains compared to the K56-2 parent strain. (C) Quantification of the zone of clearing demonstrates no significant alteration in siderophore activity in Δ*BCAL1086* and Δ*BCAL2974* mutants compared to the K56-2 parent strain. (D) Survival curve of G. mellonella infections. Data from three independent replicates of 8 to 10 larvae for each biological group are shown with the standard deviation also denoted. The Δ*BCAL1086* and Δ*BCAL2974* strains mirror the lethality of the WT and *ΔpglL amrAB*::native*-pglL-*His_10_ strains.

As changes in the glycoproteins BCAL2974 and BCAL1086 coincided with an alteration in biofilm and siderophore activity, we investigated if the loss of BCAL2974 and BCAL1086 could be responsible for defects observed in the Δ*pglL* mutant. To answer this question, *ΔBCAL2974* and *ΔBCAL1086* strains were created and assessed for their effect on biofilm production and siderophore activity, as well as virulence in G. mellonella, a phenotype previously associated with *ΔpglL* mutation (56). Both BCAL1086 and BCAL2974 have no known functions and lack homology to known domains but are present in multiple *Burkholderia* species. Assessment of 24-h static biofilm growth showed the *ΔBCAL1086* mutation had no effect on biofilm formation, while *ΔBCAL2974* resulted in a small but reproducible decrease in biofilm development. However, this effect is minimal compared to the defect observed in *ΔpglL* and *ΔcepI* mutants (Fig. 6A). The ability of *ΔBCAL1086* and *ΔBCAL2974* mutants to produce siderophores was unaffected (Fig. 6B and C). Similarly, while G. mellonella infections showed that *ΔpglL* causes reduced mortality at 48 h postinfection compared to in the K56-2 WT (*P* = 0.0015), *ΔBCAL2974*, *ΔBCAL1086*, and *ΔpglL amrAB*::native*-pglL-*His_10_ strains demonstrated wild-type levels of lethality in G. mellonella at 48 h (Fig. 6D). These results suggest that even though BCAL2974 and BCAL1086 are influenced by the loss of glycosylation, neither protein is solely responsible for the known defect observed in the Δ*pglL* mutant.

**FIG 7:**
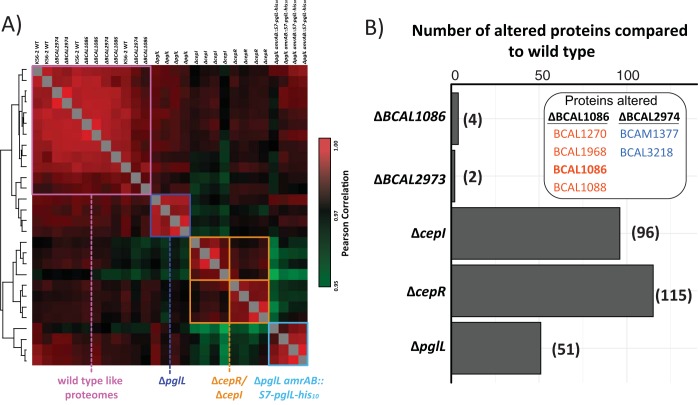
Disruption of *BCAL1086* and *BCAL2974* does affect the proteome like the Δ*pglL* mutation. (A) Pearson correlation analysis of the K56-2 WT, *ΔBCAL2974*, *ΔBCAL1086*, *ΔpglL*, *ΔcepR*, *ΔcepI*, and *ΔpglL amrAB*::S7*-pglL-*His_10_ proteomes demonstrates K56-2 WT, *ΔBCAL2974*, and *ΔBCAL1086* biological replicates cluster together, while other strains form discrete clusters. (B) Quantitative proteome analysis of Δ*BCAL1086*, Δ*BCAL2974*, Δ*cepI*, Δ*cepR*, and Δ*pglL* mutants compared to the wild type demonstrates minor proteome alterations compared to the Δ*cepI*, Δ*cepR*, and Δ*pglL* mutants.

We also investigated whether the loss of either BCAL2974 or BCAL1086 drives proteome changes. Using label-free-based quantitative proteomics, we compared the proteomes of the K56-2 WT, *ΔBCAL2974*, *ΔBCAL1086*, *ΔpglL*, *ΔcepR*, *ΔcepI*, and *ΔpglL amrAB*::S7*-pglL-*His_10_ to assess the similarity between the proteomes as well as the specific proteins affected by the loss of these proteins. Proteomic analysis led to the identification of 3,730 proteins, with 2,752 proteins quantified in at least 3 biological replicates in a single biological group (Data Set S1, tab 8). Clustering of the proteomic analysis revealed that *ΔBCAL2974* and *ΔBCAL1086* strains closely grouped with the WT strains, while the *ΔpglL*, *ΔcepR*, *ΔcepI*, and *ΔpglL amrAB*::S7*-pglL-*His_10_ strains formed discrete clusters. This macroanalysis indicated that mutations in *BCAL2974* or *BCAL1086* had a minimal effect on the proteome (Fig. 7A; Data Set S1, tabs 9 and 10). Supporting this conclusion, analysis of the specific proteins that varied between the different strains demonstrated few proteome alterations in the *ΔBCAL2974* and *ΔBCAL1086* mutants compared with the *ΔpglL*, *ΔcepR*, and *ΔcepI* mutants (Fig. 7B), with the Δ*cepR*, Δ*cepI*, and Δ*pglL* strains also demonstrating the expected similarity in their proteome changes (Fisher exact test, *ΔcepR* versus *ΔpglL* strain, *P* = 3.25 × 10^5^, and *ΔcepI* versus *ΔpglL* strain, *P* = 6.95 × 10^4^ [Data Set S1, tab 11]). Taken together, the proteome analysis results support the contention that BCAL2974 and BCAL1086 have minimal effects on the proteome and are not responsible for the broad proteomic alterations observed in the Δ*pglL* mutant.

## DISCUSSION

Although glycosylation is a common protein modification in bacterial species (49–51, 77) our understanding of how this modification influences bacterial physiology and pathogenesis is unclear. Recent insights into how glycosylation impacts bacterial proteomes have been obtained through study of the archetypical *N*-linked glycosylation system of C. jejuni (78, 79), yet it is unclear whether these observations are generalizable to other glycosylation systems such as *O*-linked glycosylation systems. Studies on the role of *N*-linked glycosylation within C. jejuni have revealed that defects associated with the loss of glycosylation stem from the loss of glycoproteins (78, 79), suggesting that *N*-linked glycosylation extends protein longevity in C. jejuni. In contrast, we find here that loss of *O*-linked glycosylation in B. cenocepacia has a more limited effect on the proteins targeted for glycosylation with only a subset of the known glycoproteins being affected by the disruption of glycosylation (Fig. 5). Therefore, the defect associated with loss of O-linked glycosylation in B. cenocepacia cannot be merely explained by protein instability. Indeed, we demonstrate that loss of glycosylation leads to changes in the expression of nonglycosylated proteins whose expression is regulated by the CepR/I regulon (Fig. 3) (39, 42, 48). Therefore, our findings uncover a previously unknown link between loss of glycosylation and alterations in pathways controlled by global transcriptional regulators.

The observation that biofilm formation is reduced in the Δ*pglL* mutant mirrors previous reports in Acinetobacter baumannii (55) and C. jejuni (63), but the link of this phenotype to alterations in regulations has not previously documented. Previous studies in B. cenocepacia have identified that not all CepR/I-regulated proteins are required for biofilm formation. However, BapA (BCAM2143) plays a major role in the formation of biofilms on abiotic surfaces, whereas the lectin complex BclACB (BCAM0184 to BCAM0186) contributes to biofilm structural development (45). Although BapA (BCAM2143) was not detected in any of our proteomic analyses, BclA and BclB (BCAM0186 and BCAM0184, respectively) were decreased in the Δ*pglL* mutant (both with a −1.0-log_2_ decrease compared with the WT; −log_10_ *P* > 3.05 [Data Set S1, tab 1]). Surprisingly, BclA and BclB increased in abundance in Δ*pglL* ΔOGC and ΔOGC strains (both 1.0 log_2_ increases compared with WT; −log_10_ *P* > 1.4 [Data Set S1, tab 1]), and these mutants formed extensive biofilms (Fig. S5B). This result agrees with recent work showing that with disruption of *BCAL3116*, the third gene in the OGC, resulted in enhanced biofilm formation (80). It also should be noted that within this study, we observed that the method of complementation of *pglL* also influenced the restoration of biofilm formation (Fig. 4A). As differences between the promoter used to drive *pglL* expression can influence some glycosylation-null phenotypes, this supports the hypothesis that *pglL* itself may be regulated under specific conditions. Concerning siderophore activity, our proteomic data reveal that siderophore-associated proteins were reduced in both Δ*pglL* and ΔOGC strains (Fig. 2), with glycosylation-null strains producing reduced zones of clearing in the CAS assays (Fig. 4B and C; Fig. S5C and D). However, the magnitude of the reduction in the CAS assays differed in the mutant, since ΔOGC and Δ*pglL* ΔOGC strains presented significantly smaller zones of clearing than the Δ*pglL* strain (Fig. S5C and D). These results highlight that although the proteome changes observed in the Δ*pglL* and ΔOGC glycosylation mutants are highly similar, they are not identical and show phenotypic differences. Therefore, a key question arising from our findings is how the loss of glycosylation alters gene regulation and whether the observed defects are simply the result of altered transcriptional control. The lack of any glycosylated signaling/receptor-associated proteins in B. cenocepacia (56) makes the identification of the link between a specific glycoprotein and transcriptional control unclear.

It is possible the observed alterations in biofilm formation and siderophore activity are not solely driven by altered CepR regulation, but also reflect additional transcriptional alterations in the glycosylation-null strains. This conclusion agrees with our observations of many differences in the abundance of transcriptional regulators in the DNA-associated proteome of glycosylation-null strains (Fig. 3C; Data Set S1, tab 4). Further, biofilm formation within B. cenocepacia is modulated by multiple transcriptional regulators (33), making CepR just one of a range of regulators that could be driving this phenotype. An additional driver of these pleiotropic effects may also be deleterious outcomes resulting from the manipulation of the *O*-linked glycosylation system. It has been suggested in C. jejuni that the disruption of glycosylation leads to undecaprenyl diphosphate decorated with *N*-linked glycan being sequestered from the general undecaprenyl diphosphate pool and that this depot effect may be a general phenomenon observed in all glycosylation mutants (64). Sequestration of undecaprenyl diphosphate was thought to drive an increase in the abundance of proteins in the nonmevalonate and undecaprenyl diphosphate biosynthesis pathways observed in glycosylation-null C. jejuni (64). However, in B. cenocepacia glycosylation mutants, we observe only minor alterations in the nonmevalonate (BCAL0802, BCAL1884, BCAL2015, BCAL2016, BCAL2085, BCAL2710, BCAM0911 and BCAM2738 [see Fig. S6A in the supplemental material]) and undecaprenyl diphosphate biosynthesis (BCAL2087 and BCAM2067 [Fig. S6B]) pathways, which argues against this phenomenon being common to all glycosylation mutants. Furthermore, the similarity of the proteome changes in the Δ*pglL*, ΔOGC, and Δ*pglL* ΔOGC strains (Fig. S2C) supports the conclusion that proteome changes are independent of the sequestration of the undecaprenyl diphosphate pool as ΔOGC and Δ*pglL* ΔOGC strains are unable build the *O*-linked glycan on undecaprenyl diphosphate. Although our proteomic analysis shows similar protein levels across glycosylation-null and -competent strains, it is important to note that we have previously shown the loss of glycosylation reduces tolerance to oxidative and osmotic stresses (65). This suggests that additional off-target effects relating to lipid-linked glycan or membrane stress may occur that are driven by changes independent of protein abundance, such as changes in protein-protein interactions, protein localization, or protein folding.

10.1128/mSphere.00660-19.7 FIG S6 Proteomics and functional analysis of strains. Comparison of Z-scored proteomic data reveals no difference between protein abundances of nonmevalonate (A) and undecaprenyl diphosphate biosynthesis (B) pathway proteins in glycosylation-null versus glycosylation-competent strains. (C) Protease probe analysis of WT, Δ*pglL* mutant, and Δ*pglL amrAB*::S7*-pglL-*His_10_ complemented strain lysates. Biological triplicates of K56-2 WT, Δ*pglL* mutant, and Δ*pglL amrAB*::S7*-pglL-*His_10_ strains were assessed using activity-based probes. No alteration in probe binding is observed, supporting similar protease activity across all strains. Download FIG S6, TIF file, 1.3 MB. Copyright © 2019 Oppy et al. 2019 Oppy et al. This content is distributed under the terms of the Creative Commons Attribution 4.0 International license.

Another explanation for the pleiotropic effects associated with loss of *O-*glycosylation could be the instability of the glycoproteins in the absence of the glycan. We identified two glycoproteins BCAL2974 and BCAL1086, both of unknown functions, which are reduced in abundance due to the loss of glycosylation. However, genetic experiments demonstrate that neither protein is responsible for the phenotypic and proteomic changes associated with loss of glycosylation (Fig. 6 and 7). Furthermore, in the case of BCAL1086, endogenous tagging and degradomic analysis confirm the loss of this protein in the *ΔpglL* background. Although these results support the breakdown of BCAL1086 as a consequence of the loss of glycosylation, an alternative explanation is that the changes in degradation arise from alterations in protease levels or activities in the *ΔpglL* mutant. Previously, we reported that *ΔpglL* results in enhanced casein proteolytic activity (65). However, our global proteome analysis shows only modest changes in protease levels. We also observed identical protease profiles from activity probe against multiple classes of protease in the wild-type, *ΔpglL*, and *ΔpglL amrAB*::S7*-pglL-*His_10_ (Fig. S6C), suggesting all of these strains have similar protease activities. More importantly, aside from glycoproteins BCAL2974 and BCAL1086, the other proteins targeted for glycosylation remain consistently stable in the glycosylation-defective mutants. Although 23 glycoproteins are known in B. cenocepacia, additional glycoproteins may also exist that were missed in the initial characterization of B. cenocepacia glycoproteome. Regardless, although loss of glycosylation may affect the stability of some glycoproteins, the pleiotropic effect found in the glycosylation mutants cannot be explained by alterations in protein degradation.

In summary, this work provides a global analysis of the effect of *O*-linked glycosylation on B. cenocepacia traits. The application of quantitative proteomics enabled the assessment of nearly half the predicted proteome of B. cenocepacia K56-2 and revealed a previously unknown link between *O*-linked glycosylation and transcriptional alterations. The alteration in known transcriptional regulators, such as CepR, as well as its associated phenotypes, supports a model in which the defects observed for glycosylation-null strains arise from transcriptional changes and not from the direct result of glycosylation loss *per se*. This work challenges the idea that loss of glycosylation solely affects the stability and activity of the glycoproteome and instead shows that glycosylation can influence the bacterial transcriptional profile and broader proteome.

## MATERIALS AND METHODS

### Bacterial strains and growth conditions.

The strains and plasmids used in this study are listed in Tables 1 and 2, respectively. Strains of Escherichia coli and B. cenocepacia were grown at 37°C in Luria-Bertani (LB) medium. When required, antibiotics were added to the following final concentrations: 50 μg/ml trimethoprim for E. coli and 100 μg/ml for B. cenocepacia, 20 μg/ml tetracycline for E. coli and 150 μg/ml for B. cenocepacia, and 40 μg/ml kanamycin for E. coli. Ampicillin was used at 100 μg/ml and polymyxin B at 25 μg/ml for triparental mating to select against donor and helper E. coli strains. Antibiotics were purchased from Thermo Fisher Scientific, while all other chemicals unless otherwise stated were provided by Sigma-Aldrich.

**TABLE 1:** Strains used in this study

Strain	Description	Source
E. coli		
DH5α	F^−^ ϕ80*lacZ*ΔM15 *endA1 recA1 hsdR17*(r_K_^−^ m_K_^+^) *phoA supE44 thi-1* Δ*gyrA96* (Δ*lacZYA-argF*)*U169 relA1F* λ^−^	Invitrogen
PIR2	F^−^ Δ*lac169 rpoS*(Am) *robA1 creC510 hsdR514 endA recA1 uidA*(ΔMluI)::*pir-116*	Thermo Scientific
B. cenocepacia		
K56-2	Clinical isolate of the ET12 lineage^*a*^	Canadian B. cepacia Research and Referral Repository
K56-2 Δ*pglL*	Δ*pglL* (*BCAL0960*) derivative of K56-2 created using pYM8	This study
K56-2 ΔOGC	ΔOGC (*BCAL3114*–*BCAL3118*) derivative of K56-2 created using pGPi-SceI-OGC	This study
K56-2 Δ*pglL amrAB*::S7- *pglL*-His_10_	*amrAB*::S7-*pglL*-His_10_ chromosomal complement derivative of Δ*pglL* (*BCAL0960*) mutant created using pMH447-S7-*pglL*-His_10_, gentamicin-sensitive strain	This study
K56-2 Δ*pglL amrAB*::native -*pglL*-His_10_	*amrAB*::native *pglL* promoter-*pglL*-His_10_ chromosomal complement derivative of Δ*pglL* (*BCAL0960*) mutant created using pMH447-native-*pglL*-His_10_, gentamicin-sensitive strain	This study
K56-2 *BCAL1086*-His_10_	Chromosomally tagged *BCAL1086* with a C-terminal His_10_	This study
K56-2 Δ*pglL BCAL1086*-His_10_	Δ*pglL* (*BCAL0960*) mutant derivative of K56-2, chromosomally tagged *BCAL1086* with a C-terminal His_10_	This study
K56-2 Δ*pglL amrAB*::S7- *pglL*-His_10_ *BCAL1086*-His_10_	*amrAB*::S7-*pglL*-His_10_ chromosomal complement derivative of Δ*pglL* (*BCAL0960*) mutant with a chromosomally tagged *BCAL1086* with a C-terminal His_10_	This study
K56-2 Δ*BCAL1086*	Δ*BCAL1086* derivative of K56-2 created using pGPi-SceI-*BCAL1086*	This study
K56-2 Δ*BCAL2974*	Δ*BCAL2974* derivative of K56-2 created using pGPi-SceI-*BCAL2974*	This study
K56-2 Δ*cepR*	Δ*cepR* derivative of K56-2 created using pGPi-SceI-*cepR*	This study
K56-2 Δ*cepI*	Δ*cepI* derivative of K56-2 created using pGPi-SceI-*cepI*	This study

a See references 4 and 104 for details.

**TABLE 2:** Plasmids used in this study

Plasmid	Description	Reference(s)
pRK2013	*ori_colE1_*, RK2 derivative, Kan^r^ *mob*^+^ *tra*^+^	105
pGPI-SceI	*oriR6K* mob^+^ ΩTp^r^, including ISce-I restriction site	83
pDAI-SceI-SacB	*ori*_pBBR1_ Tet^r^ P_dhfr_ *mob*^+^, expressing ISce-I and negative selection marker SacB	83, 106
pMH447	pGPI-SceI with fragments flanking Δ*amrAB* (*BCAL1674*–*BCAL1675*)	106
pYM8	pGPI-SceI with fragments flanking *pglL* (*BCAL0960*)	65
pGPI-SceI-OGC	pGPI-SceI with fragments flanking OGC (*BCAL3114*–*BCAL3118*)	This study
pMH447-S7*-pglL-*His_10_	pMH447 with S7 promoter driving expression of *pglL-*His_10_ from Met11 of open reading frame *BCAL0960*	This study
pMH447-native-*pglL*-His_10_	pMH447 with native *pglL*-His_10_ promoter driving expression of *pglL*	This study
pGPI-SceI-*BCAL1086*	pGPI-SceI with fragments flanking *BCAL1086* to generate Δ*BCAL1086* mutant	This study
pGPI-SceI-*BCAL2974*	pGPI-SceI with fragments flanking *BCAL2974* to generate Δ*BCAL2874* mutant	This study
pGPI-SceI-*cepR*	pGPI-SceI with fragments flanking *cepR* (*BCAM1868*) to generate Δ*cepR* mutant	This study
pGPI-SceI-*cepI*	pGPI-SceI with fragments flanking *cepI* (*BCAM1870*) to generate Δ*cepI* mutant	This study
pGPI-SceI-*BCAL1086*-His_10_	pGPI-SceI with fragments flanking *BCAL1086* to generate *BCAL1086*-His_10_ mutant	This study
pKM4	Tp^r^ pMLBad‐based plasmid containing C- terminal His_6_‐tagged DsbA1 from Neisseria meningitidis MC58	66
pMS402	Promoterless *luxCDABE* promoter reporter plasmid, Kan^r^ Tp^r^	92
pPromcepR	*cepR*::*luxCDABE* transcriptional fusion in pMS402, Kan^r^ Tp^r^	69
pCP300	*cepI*::*luxCDABE* transcriptional fusion in pMS402, Kan^r^ Tp^r^	72

### Recombinant DNA methods.

The oligonucleotides used in this study are listed in Table 3. DNA ligations, restriction endonuclease digestions, and agarose gel electrophoresis were performed using standard molecular biology techniques (81), with Gibson assembly undertaken according to published protocols (82). All restriction enzymes, T4 DNA ligase, and Gibson master mix were used as recommended by the manufacturer (New England Biolabs). E. coli PIR2 and DH5α cells were transformed using heat shock-based transformation. PCR amplifications were carried out using either Phusion DNA (Thermo Fisher Scientific) or *Pfu* Ultra II (Agilent) polymerases were used according to the manufacturer’s recommendations with the addition of 2.5% dimethyl sulfoxide (DMSO) for the amplification of B. cenocepacia DNA due to its high GC content. DNA isolation, PCR recoveries, and restriction digest purifications were performed using the genomic DNA cleanup kit (Zmyo Research, CA) or Wizard SV gel and PCR cleanup system (Promega). Colony and screening PCRs were performed using GoTaq *Taq* polymerase (Qiagen) supplemented with 10% DMSO when screening B. cenocepacia. All constructs in Table 2 were confirmed by Sanger sequencing undertaken at the Australian Genome Research Facility (Melbourne, Australia).

**TABLE 3:** Primers used in this study

Primer	Sequence^*a*^	Description	Restriction site^*b*^
NS01	AAATCTAGAGTGACGACGATGCACGAAT	*BCAL3114* (*ogcX*) forward	XbaI
NS02	AAACTCGAGAAATTAATTTAATTGATCTGGGTGAGCCGTTC	*BCAL3114* (*ogcX*) reverse	XhoI
NS03	AAACTCGAGAATAAGCTTCATCGTCTCCCTGCT	*BCAL3118* (*ogcI*) forward	XhoI
NS04	AAACCCGGGGAAGCAGGTCTCGAAGATCG	*BCAL3118* (*ogcI*) reverse	SmaI
NS05	AAACCCGGGGCCTACGTGATCTTCCACGA	*BCAL0230* (S7) promoter forward for *pglL* cloning	SmaI
NS06	CGAACGGGAAAAAGTAGAAGGCATGATTCTTCCTTTACTTGTTC AGTTGGAGC	*BCAL0230* (S7) promoter reverse for *pglL* cloning from Met11	—
NS07	GCTCCAACTGAACAAGTAAAGGAAGAATCATGCCTTCTACTTTTT CCCGTTCG	*BCAL0960* (*pglL*) forward amplifying from Met11	—
NS08	AAACCCGGGTCAGTGGTGGTGGTGGTGGTGGTGGTGGTGGTGA TCCGAATCGTCGTCGTCCG	*BCAL0960* (*pglL*) reverse with His_10_ tag	SmaI
NS09	GGAATTTCACGACATGGCCCGCAAGACCCTTCACGCTGATCGAACTGAT	*BCAL0960* (*pglL*) with native promoter forward	—
NS10	CCGGTGCTTGATGGCGAGCGATTCTTCCCTCAGTGGTGGTGGTG GTGGTGGTGGTGGTGGTGATCCGAATCGTCGTCGTCCG	*BCAL0960* (*pglL*) with native promoter reverse	—
NS11	TTTTGAATTCGCGTTCGAGGTACCAGTCC	*BCAM1868* (*cepR*) upstream forward	EcoRI
NS12	TTTTGTCGACCCCGAGCCGCTTGAATAG	*BCAM1868* (*cepR*) upstream reverse	SalI
NS13	TTTTGTCGACTCCACGTGAACAACATCCTC	*BCAM1868* (*cepR*) downstream forward	SalI
NS14	TTTTTCTAGACTGCTCACCAATACGGTGCT	*BCAM1868* (*cepR*) downstream reverse	XbaI
NS15	TTTATCTAGACTGGGACTGGTACCTCGAAC	*BCAM1870* (*cepI*) upstream forward	XbaI
NS16	TTTTCTCGAGAGGTCTGCATGGATGTCCTC	*BCAM1870* (*cepI*) upstream reverse	XhoI
NS17	TTTTCTCGAGAGGTAGATGGGCGTCTGGT	*BCAM1870* (*cepI*) downstream forward	XhoI
NS18	TTTAGAATTCCTGCATCGTCAGGTCGTG	*BCAM1870* (*cepI*) downstream reverse	EcoRI
NS19	AAAACTCGAGCTATCGATCGCGTCTTCGTTAC	*BCAL2974* upstream reverse	XhoI
NS20	AAAATCTAGAGCCACGTGCTGATTCATCT	*BCAL2974* upstream forward	XbaI
NS21	AAAAGAATTCCTGGATCGCTTCCGGATAAT	*BCAL2974* downstream forward	EcoRI
NS22	AAAACTCGAGGAAATACCAGGGCAGCAAGA	*BCAL2974* downstream reverse	XhoI
NS23	AAAACTCGAGGCAAGAAACTCCAGCAGTGG	*BCAL1086* downstream forward	XhoI
NS24	AAAAGAATTCATTCAATGAACGTTGCGTCA	*BCAL1086* downstream reverse	EcoRI
NS25	AAAATCTAGATGCGTACGACGAACGAAG	*BCAL1086* upstream forward	XbaI
NS26	AAAACTCGAGCTACATGTTCATCAGCAATCTCCGG	*BCAL1086* upstream reverse	XhoI
NS27	AAAATCTAGATGCGATTGAATCAGACGAAT	*BCAL1086* His_10_ tagging upstream forward	XbaI
NS28	TCAGTGGTGGTGGTGGTGGTGGTGGTGGTGGTGTTCACCGCT CGCGGGTTTGGCC	*BCAL1086* His_10_ tagging upstream reverse	—
NS29	CCACCACCACCACCACCACTGACGCGGGCTTGCACGATCCGC	*BCAL1086* His_10_ tagging downstream forward	—
NS30	AAAATCTAGACTCCAGATCCAGCATGTCG	*BCAL1086* His_10_ tagging downstream reverse	XbaI

a Restriction sites are underlined when present.

b —, primer for Gibson assembly or overlap PCR.

### Construction of unmarked deletion mutants, endogenous tagged BCAL1086, and complementation with *pglL-*His_10_.

Deletions and endogenous tagging of BCAL1086 were undertaken using the approach of Flannagan et al. for the construction of unmarked, nonpolar deletions in B. cenocepacia K56-2 (83). Chromosomal complements of *pglL* were generated by introducing *pglL-*His_10_ under the control of the B. cenocepacia S7 promoter (*P*_S7_) or the native *pglL* promoter (Ppgl; 660 bp upstream of PglL) inserted into *amrAB* using the pMH447 (23) derivative plasmids (Table 2) according to the protocol of Aubert et al. (84).

### Protein manipulation and immunoblotting.

Bacterial whole-cell lysates were prepared from overnight LB cultures of B. cenocepacia strains. One milliliter of bacteria at an optical density at 600 nm (OD_600_) of 1.0 were pelleted, then resuspended in a mixture of 4% sodium dodecyl sulfate (SDS), 100 mM Tris (pH 8.0), and 20 mM dithiothreitol (DTT) and boiled at 95°C with shaking at 2,000 rpm for 10 min. Samples were then mixed with Laemmli loading buffer (24.8 mM Tris, 10 mM glycerol, 0.5% [wt/vol] SDS, 3.6 mM β-mercaptoethanol, and 0.001% [wt/vol] bromophenol blue (pH 6.8), final concentration) and heated for a further 5 min at 95°C. Lysates were then subjected to SDS-PAGE using precast 4 to 12% gels (Invitrogen) and transferred to nitrocellulose membranes. Membranes were blocked for 1 h in 5% skim milk in TBS-T (20 mM Tris, 150 mM NaCl and 0.1% Tween 20) and then incubated for at least 16 h at 4°C with either mouse monoclonal anti-His (AD1.1.10, 1:2,000 [AbD Serotech]) or mouse anti-RNA pol (4RA2, 1:5,000 [Neoclone]). Proteins were detected using anti-mouse IgG horseradish peroxidase (HRP)-conjugated secondary antibodies (1:3,000 [Perkin-Elmer catalog no. NEF822001EA]) and developed with Clarity Western ECL (enhanced chemiluminescence) substrate (Bio-Rad). All antibodies were diluted in TBS-T with 1% bovine serum albumin (BSA [Sigma-Aldrich]). Images were obtained using an MFChemiBis imaging station (DNR Bio-Imaging Systems) or an Amersham imager 600 (GE Life Sciences).

### Proteomic analysis.

Whole-proteome sample preparation was undertaken as previously described (65), while peptidomic and DNA binding proteome analysis were undertaken according to the approaches of Parker et al. (76) and Qin et al. (85), respectively. For nonpeptidomic samples, isolated protein preparations were digested as previously described (86) and cleaned up using homemade stage tips according to the protocol of Ishihama and Rappsilber (87, 88). Peptidomic samples were cleaned up using commercial tC_18_ columns (Waters). Purified peptides were resuspended in buffer A* (2% acetonitrile [ACN], 0.1% trifluoroacetic acid) and separated using a two-column chromatography setup comprising a PepMap100 C_18_ 20-mm by 75-μm trap and a PepMap C_18_ 500-mm by 75-μm analytical column (Thermo Scientific). Data were acquired on either an Orbitrap Elite mass spectrometer (Thermo Scientific), an Orbitrap Fusion Lumos Tribrid mass spectrometer (Thermo Scientific), or a Q-exactive plus mass spectrometer (Thermo Scientific) and processed using MaxQuant (v1.5.5.1 or 1.5.3.30 [89]). Database searching was carried out against the reference B. cenocepacia strain J2315 (https://www.uniprot.org/proteomes/UP000001035) and the K56-2 Valvano (90) (http://www.uniprot.org/taxonomy/985076) proteomes. Proteomic data sets have been deposited into the ProteomeXchange Consortium via the PRIDE (91) partner repository. A complete description of each PRIDE data set is provided in Table 4. A complete description of all proteomic-associated methods is provided in Text S1 in the supplemental material.

**TABLE 4:** Description of proteomic experiments within PRIDE repository^*a*^

PRIDE accession no.	Title	Description
PXD014614	Peptidomic analysis of B. cenocepacia strains	Comparison of endogenous peptide pool in B. cenocepacia K56-2 strains to identify evidence for glycoprotein degradation in the absence of glycosylation; strains used are K56-2 WT, Δ*pglL* mutant, and Δ*pglL amrAB*::S7*-pglL-*His_10_ complemented strain; LFQ-based quantification undertaken using Maxquant with 4 biologicals of each strain type
PXD014581	LFQ B. cenocepacia mutant comparison	Comparison of multiple B. cenocepacia K56-2 mutants to assess proteome changes; 7-strain comparison between the K56-2 WT, Δ*pglL*, Δ*BCAL*1086, Δ*BCAL*2974, Δ*cepI*, Δ*cepR* and Δ*pglL* mutants, and *amrAB*::S7*-pglL-*His_10_ complemented strain; LFQ-based quantification undertaken using Maxquant with 5 biologicals of each strain type
PXD014516	LFQ B. cenocepacia Δ*pglL* mutant comparison	Characterization of the effect of *pglL* mutants and complement in B. cenocepacia K56-2; 6-strain comparison of K56-2 WT, Δ*pglL* (independent mutant 1), Δ*pglL* (independent mutant 2), Δ*pglL* (independent mutant 1) *amrAB*::native-*pglL-*His_10_, Δ*pglL* (independent mutant 1) *amrAB*::S7*-pglL-*His_10_, and Δ*pglL* (independent mutant 2) *amrAB*::native-*pglL-*His_10_ strains; LFQ- based quantification with 4 biologicals of each strain type
PXD014429	LFQ B. cenocepacia glycosylation mutant comparison	Characterization of effect of glycosylation disruption in *B.* *cenocepacia* K56-2; 5-strain comparison of K56-2 WT, Δ*pglL*, ΔOGC, Δ*pglL* ΔOGC, and Δ*pglL amrAB*::S7*-pglL-*His_10_ strains; LFQ- based quantification undertaken using Maxquant with 5 biologicals of each strain type
PXD014700	LFQ B. cenocepacia comparison of DNA binding proteome	Comparison of alterations in the DNA-bound proteome of *B.* *cenocepacia* K56-2 mutants. DDA experiments undertaken using 4 strains; K56-2 WT, Δ*pglL*, ΔOGC, and Δ*pglL amrAB*::S7*-pglL-*His_10_ strains used for DDA experiments, while K56-2 WT, Δ*pglL*, and Δ*pglL amrAB*::*S7-pglL-*His_10_ strains used for DIA experiments; LFQ-based quantification undertaken using Maxquant with four biologicals of each strain type

a All proteomic data in this study have been uploaded to the PRIDE proteomic repository and are accessible through the corresponding accession numbers. LFQ, label-free quantification; DDA, data-dependent acquisition; DIA, data-independent acquisition.

10.1128/mSphere.00660-19.1 TEXT S1 Complete proteomic methods. Download Text S1, DOCX file, 0.1 MB. Copyright © 2019 Oppy et al. 2019 Oppy et al. This content is distributed under the terms of the Creative Commons Attribution 4.0 International license.

### Motility assays.

Motility assays were conducted using semisolid motility agar consisting of LB infusion medium supplemented with 0.3% agar as previously described (56). Plates were inoculated using 2 μl of standardized (OD_600_ of 0.5) overnight cultures of each strain. Motility zones were measured after 48 h of incubation at 37°C. Experiments were carried out in triplicate with 3 biological replicates of each strain.

### Transcriptional analysis by luminescence assays.

To assess transcriptional changes in CepR and CepI, *luxCDABE* reporter assays were performed using the B. cenocepacia K56‐2 wild‐type (WT), *ΔpglL*, *Δ*OGC, and *ΔpglL amrAB*::S7*-pglL-*His_10_ strains containing pCP300 (CepI promoter *luxCDABE* reporter [72]), pPromcepR (CepR promoter *luxCDABE* reporter [69]) or pMS402 (promoterless *luxCDABE* reporter [92]) as a negative control. Overnight cultures were diluted to an OD_600_ of 1.0, and 2 μl was inoculated into 200 μl LB supplemented with 100 μg/ml trimethoprim in black, clear‐bottom 96‐well microplates (minimum of eight technical replicates per independent biological replicate). The OD_600_ and relative luminescence were measured using a CLARIOstar plate reader at 10-min intervals for 24 h. Experiments assessing the effect of C_8_-HSL additions on CepR and CepI transcription were performed according to Le Guillouzer et al. (93). Briefly, cultures were supplemented with C_8_-HSL (Sigma-Aldrich) resuspended in acetonitrile (10 μM final concentration) and added to cultures with acetonitrile added alone used as a negative control. Plates were incubated at 37°C with shaking at 200 rpm between measurements, with each assay undertaken 3 independent times on separate days. The resulting outputs were visualized using R (https://www.r-project.org/).

### Biofilm assay.

Biofilm assays were performed according to previous reports (26, 94, 95) using protocols based on the approach of O’Toole (96). B. cenocepacia strains were grown overnight at 37°C and adjusted to an OD_600_ of 1.0. Ten microliters of these suspensions was inoculated into 990 μl of LB supplemented with 0.5% (wt/vol) Casamino Acids, and 100 μl was added into 96-well microtiter plates (Corning Life Sciences [a minimum of eight technical replicates per independent biological replicate]). Microtiter plates were incubated at 37°C for 24 h in a closed humidified plastic container. The plates were then washed with phosphate-buffered saline (PBS) to remove planktonic cells then stained for 15 min with 125 μl of 1% (wt/vol) crystal violet. Excess crystal violet was removed with two washes of PBS and 200 μl of 33% (vol/vol) acetic acid was added for 15 min to release the stain. The resuspended stain was transferred to a new plate and measured on a CLARIOstar plate reader measuring the absorbance of the resulting solution at 595 nm. Three independent assays were undertaken on separate days.

### Galleria mellonella infection assays.

Infection of *G. mellonella* larvae was undertaken using the approach of Seed and Dennis (97) with minor modifications. B. cenocepacia strains were grown overnight at 37°C and adjusted to an OD_600_ of 1.0, equivalent to 2 × 10^9^ CFU/ml. Strains were diluted with PBS to 4 × 10^5^ CFU/ml, with serial dilution plates undertaken to confirm inoculum levels. For each strain, 2,000 CFU in 5 μl was injected in the right proleg of the G. mellonella larvae. Three independent challenges were performed with each strain injected into 8 to 10 G. mellonella larvae. For each independent challenge, 8 control larvae were injected with 5 μl PBS. Postinfection, G. mellonella larvae were placed in 12-well tissue culture plates and incubated in the dark at 30°C. The number of dead larvae was scored at 24, 48, and 72 h after infection, with death of the larvae determined by loss of responsiveness to touch. The results visualized using R (https://www.r-project.org/), and statistical analysis of survival curves was undertaken with the survminer package (version 0.4.5).

### CAS siderophore assays.

Alterations in activities of siderophores were assessed using the chrome azurol S (CAS) assay as previously described (98, 99). Ten microliters of adjusted bacterial culture at an OD_600_ of 1.0 was spotted on CAS agar plates and incubated at 37°C for 24 h. The diameter of the zone of discoloration from the removal of iron from the CAS dye complex was measured. Experiments were carried out with at least 3 biological replicates in technical triplicate.

### Protease activity-based probes.

K56-2 WT, *ΔpglL*, and *ΔpglL amrAB*::S7*-pglL-*His_10_ strains were grown overnight on confluent LB plates. Plates were flooded with 5 ml of prechilled sterile PBS, and colonies were removed with a cell scraper. Cells were washed 3 times in chilled PBS and resuspend in 40 mM Tris–150 mM NaCl (pH 7.8) and then lysed by sonication. Samples were clarified by centrifugation at 10,000 × *g* for 10 min at 4°C, and samples were diluted to a total concentration of 4 mg/ml. Reactivity to three classes of activity-based probes was assessed using PK-DPP (Cy5-tagged probe for trypsin-like proteases [100]), PK105b (Cy5-tagged probe for elastase-like proteases [101]), PK101 (biotin-tagged probe for elastase-like proteases [102]), and FP-biotin (biotin-tagged probe for serine hydrolases [103]), which were added at 1.3 μM from a 100× DMSO stock. Untreated control samples were prepared in parallel and left untreated to allow the assessment of autofluorescence and endogenous biotinylation in lysates. Samples were incubated at 37˚C for 15 min to allow labeling and then quenched by the addition of Laemmli sample buffer. Samples were then boiled, and proteins were resolved on a 15% SDS-PAGE gel. For Cy5-tagged probes, labeling was detected by directly scanning the gel for Cy5 fluorescence using a Typhoon 5 flatbed laser scanner (GE Healthcare). For FP-biotin, proteins were transferred to nitrocellulose and the membrane was incubated with streptavidin-Alexa Fluor 647 at 4°C overnight. Following three washes with PBS containing 0.05% Tween 20, the membrane was scanned on the Typhoon 5 in the Cy5 channel. Experiments were carried out in biological triplicate. All probes were synthesized in-house by the Edgington-Mitchell Laboratory according to published methods, with the exception of FP-biotin, which was purchased from Santa Cruz Biotechnology.

### Data availability.

Proteomic data sets have been deposited into the ProteomeXchange Consortium via the PRIDE (91) partner repository with the data set identifiers PXD014429, PXD014516, PXD014581, PXD014614, and PXD014700.

10.1128/mSphere.00660-19.8 TABLE S1 Compiled virulence-associated genes in B. cenocepacia J2315. J2315 genes defined as virulence associated used for enrichment analysis are provided with PMID references denoting the source of the assignment as a virulence factor. Download Table S1, XLSX file, 0.1 MB. Copyright © 2019 Oppy et al. 2019 Oppy et al. This content is distributed under the terms of the Creative Commons Attribution 4.0 International license.
