# Supplementary figures and images for: Loss of O-Linked Protein Glycosylation in Burkholderia cenocepacia Impairs Biofilm Formation and Siderophore Activity and Alters Transcriptional Regulators
Source: mSphere. 2019 Nov 13;4(6):e00660-19. doi: 10.1128/mSphere.00660-19 (PMC6854043; doi:10.1128/mSphere.00660-19)

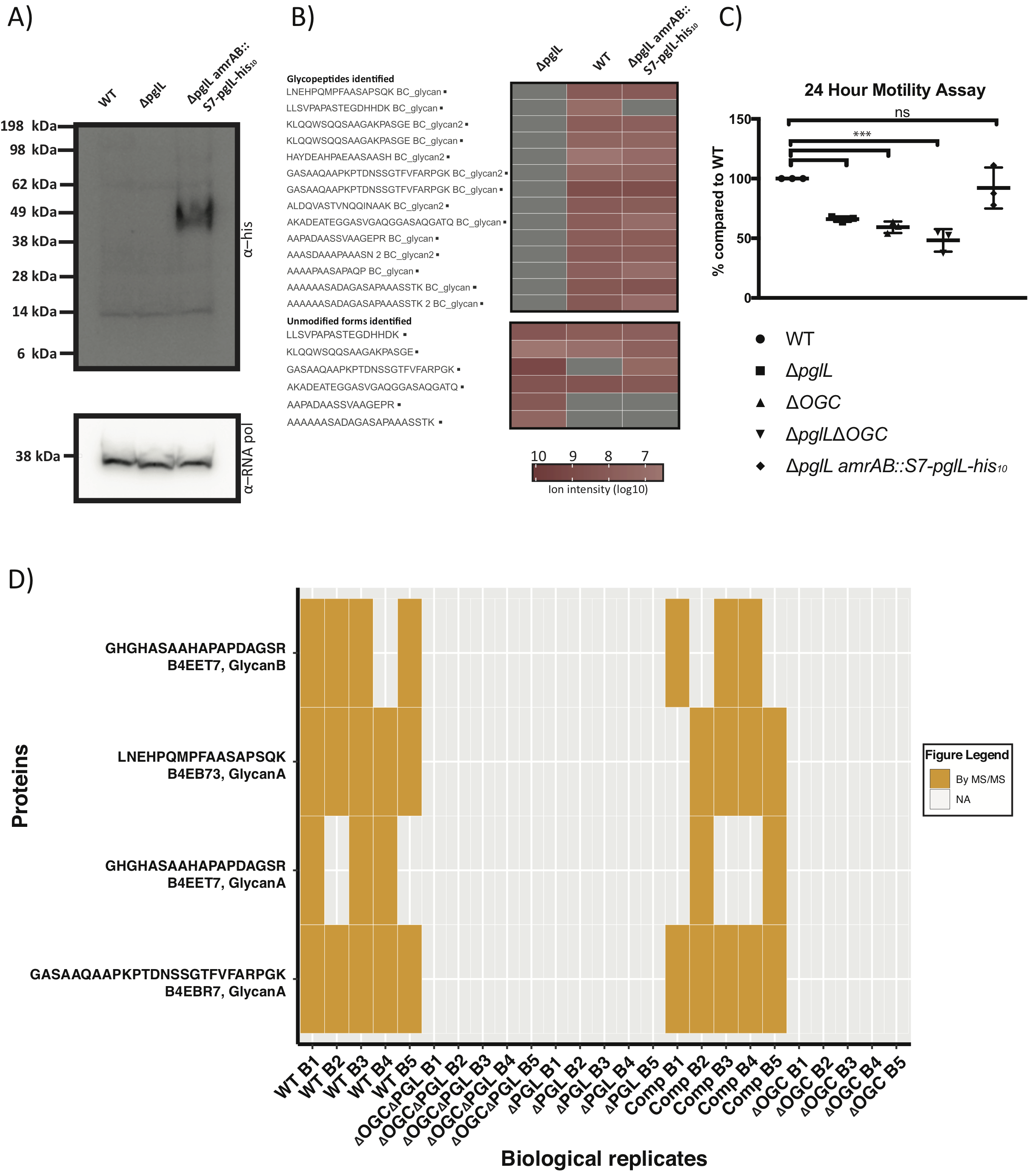

Supplement: FIG S1 [file mSphere.00660-19-sf001.tif]

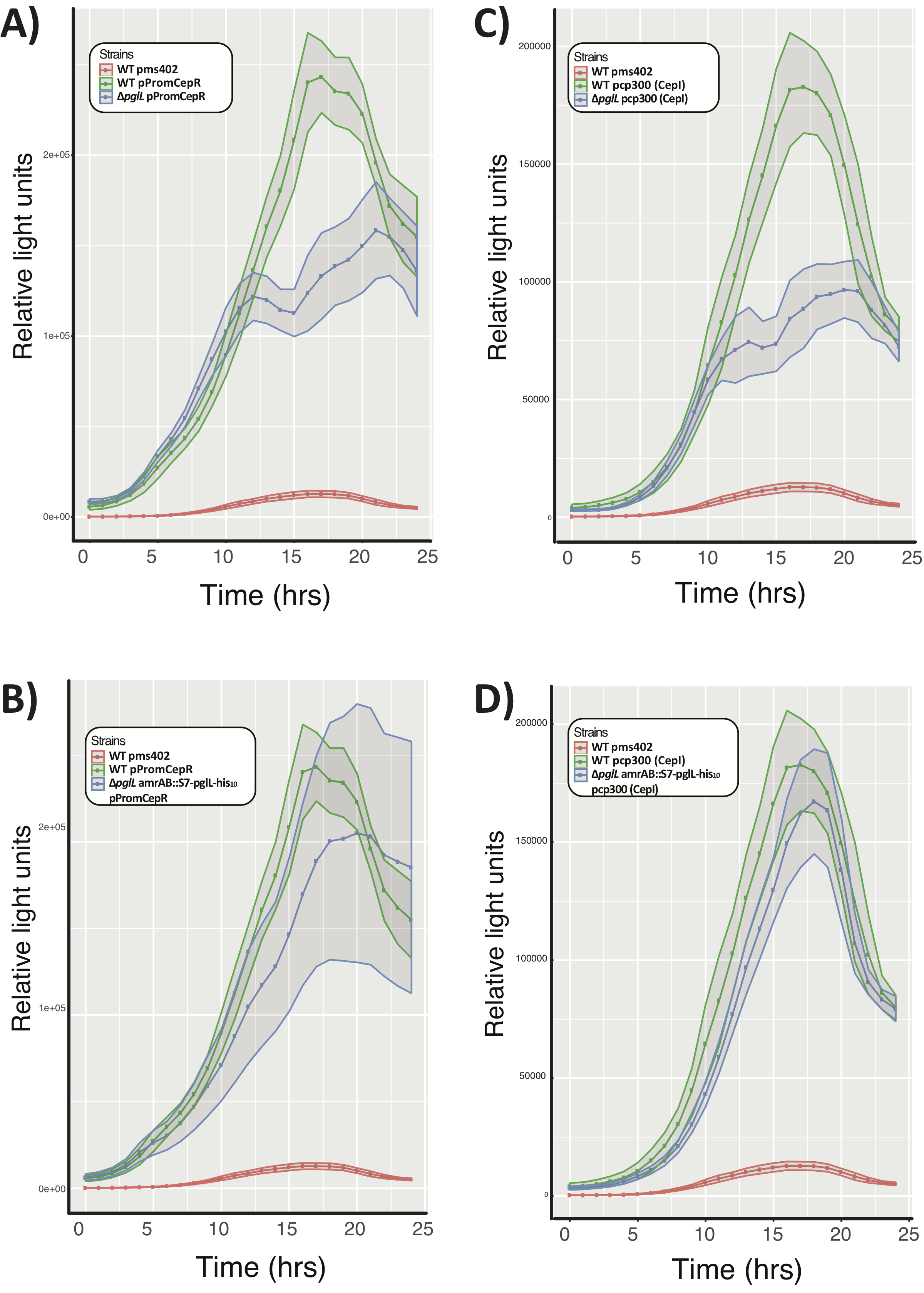

Supplement: FIG S3 [file mSphere.00660-19-sf003.tif]

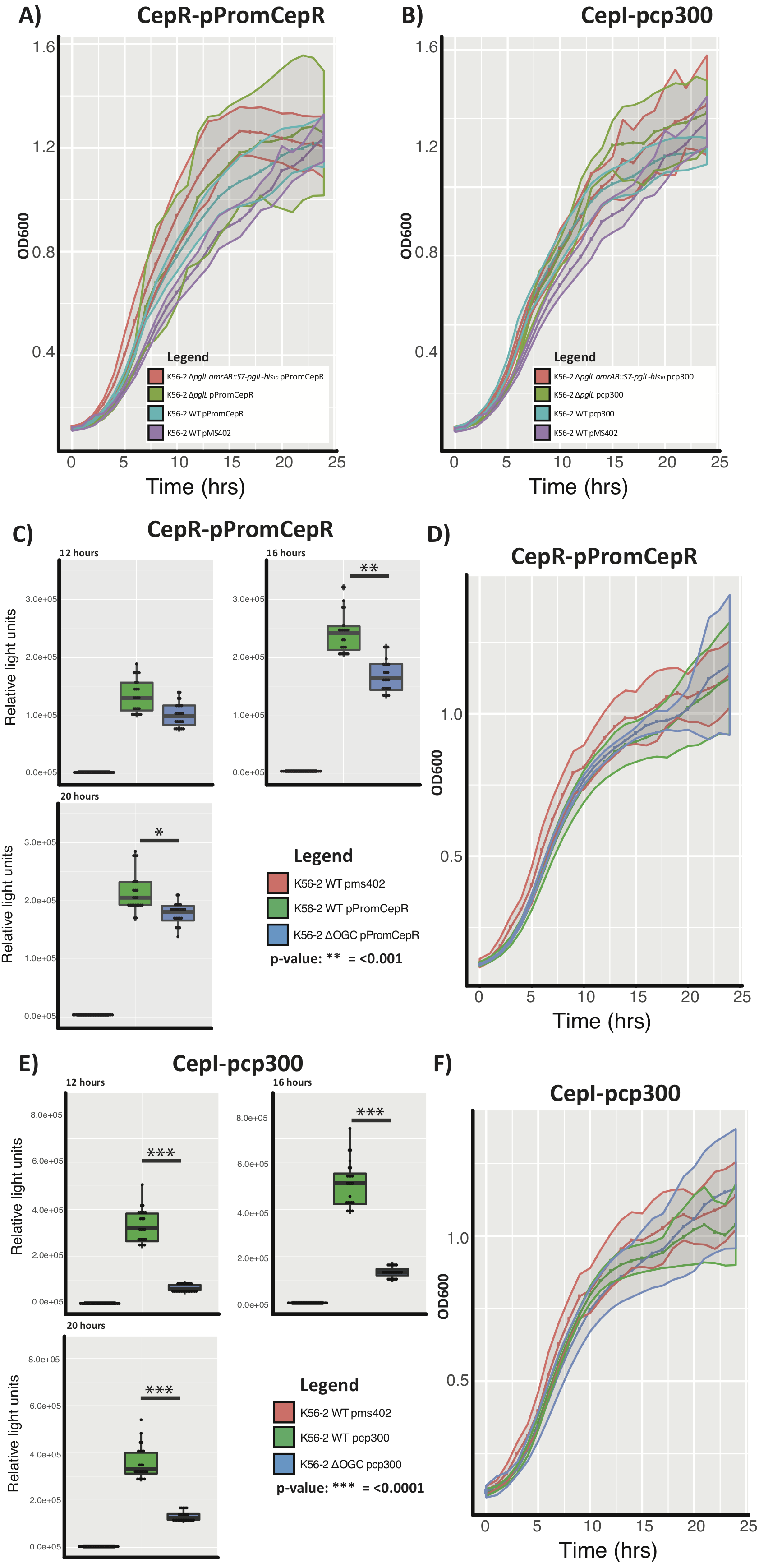

Supplement: FIG S4 [file mSphere.00660-19-sf004.tif]

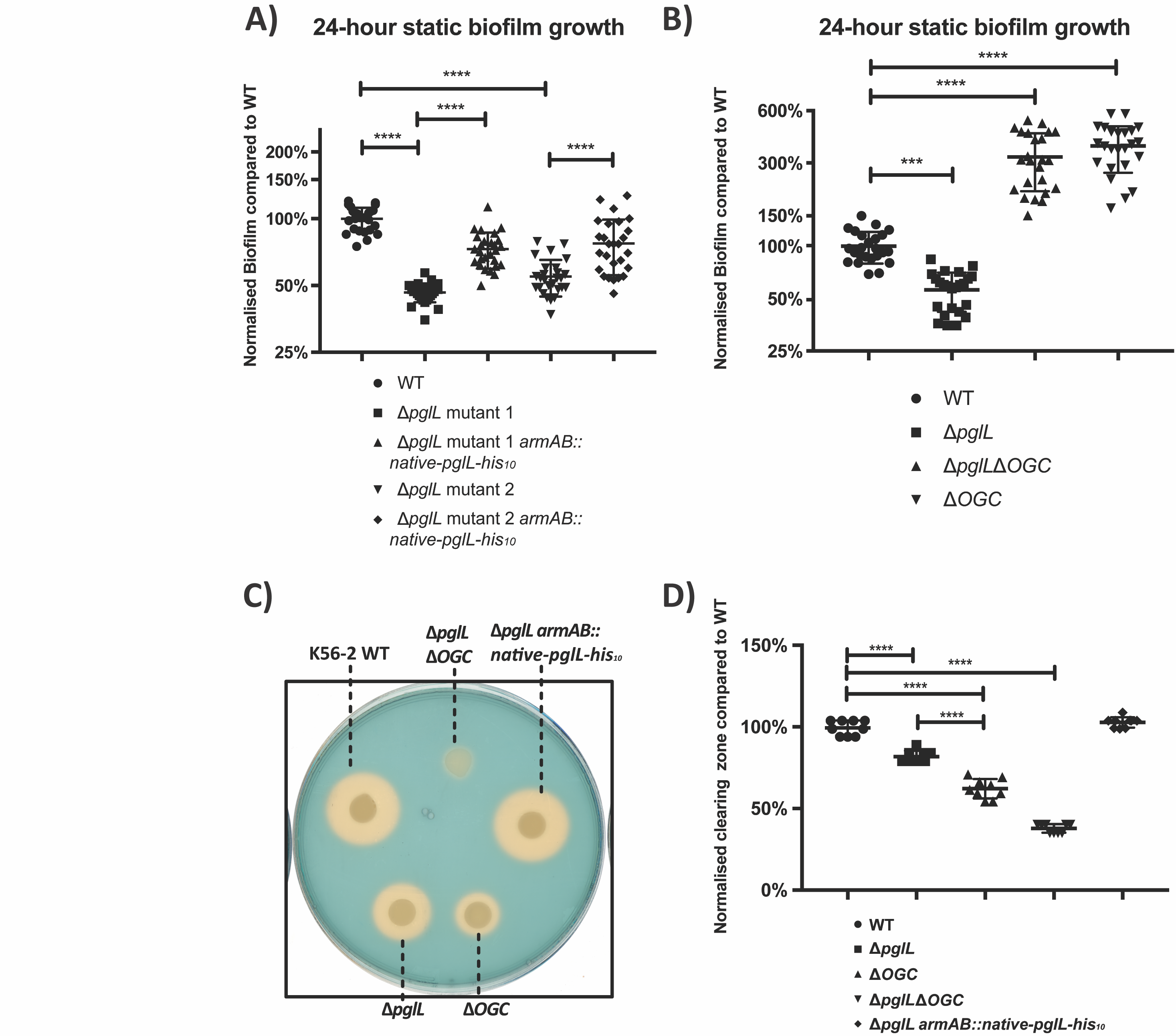

Supplement: FIG S5 [file mSphere.00660-19-sf005.tif]

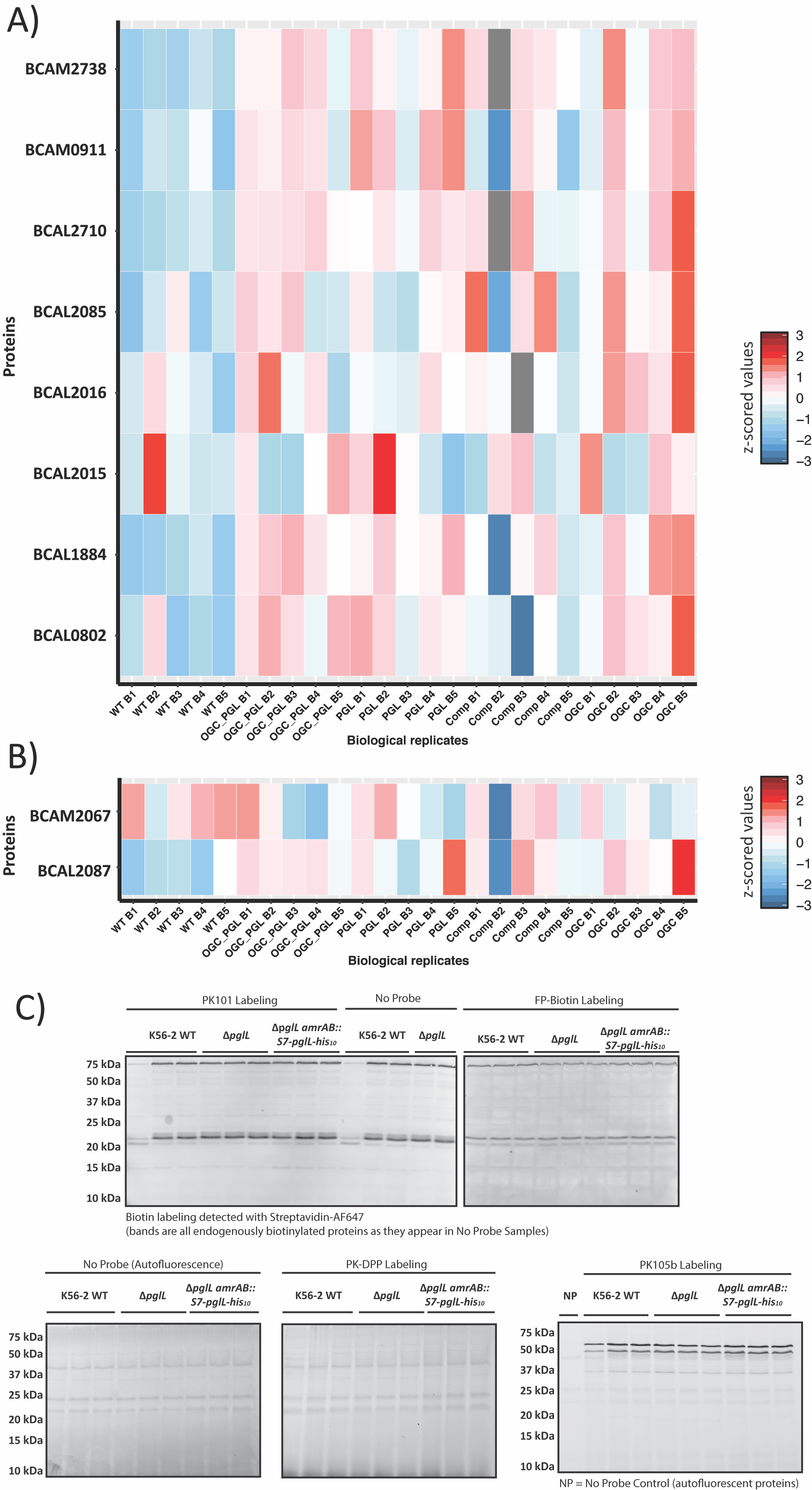

Supplement: FIG S6 [file mSphere.00660-19-sf006.tif]
