# Supplementary material for: Loss of O-Linked Protein Glycosylation in Burkholderia cenocepacia Impairs Biofilm Formation and Siderophore Activity and Alters Transcriptional Regulators
Source: mSphere. 2019 Nov 13;4(6):e00660-19. doi: 10.1128/mSphere.00660-19 (PMC6854043; doi:10.1128/mSphere.00660-19)
Supplement: TEXT S1 [file mSphere.00660-19-s0001.docx]

**Loss of *O*-linked protein glycosylation in *Burkholderia cenocepacia* impairs biofilm formation, siderophore activity** **and alters transcriptional regulators.**

**(Supplementary Methods: Complete Proteomic Methods)**

Cameron C. Oppy^1,2*^, Leila Jebeli^1*^, Miku Kuba^1^, Clare V. Oates^1^, Richard Strugnell^1^, Laura E. Edgington-Mitchell^2-4^, Miguel A. Valvano^5^**^,^**^6^, Elizabeth L. Hartland^1,7,8^, Hayley J. Newton^1^, Nichollas E. Scott^1^

^1^Department of Microbiology and Immunology, University of Melbourne at the Peter Doherty Institute for Infection and Immunity, Melbourne 3000, Australia

^2^Bio21 Molecular Science and Biotechnology Institute, University of Melbourne, Victoria 3010, Australia

^3^Drug Discovery Biology, Monash Institute of Pharmaceutical Sciences, Monash University, Parkville, VIC, Australia

^4^Department of Oral and Maxillofacial Surgery, New York University College of Dentistry, Bluestone Center for Clinical Research, New York, New York, USA

^5^Wellcome-Wolfson Institute for Experimental Medicine, Queen's University Belfast, Belfast, BT97BL, United Kingdom

^6^Department of Microbiology and Immunology, University of Western Ontario, London, ON, N6A 5C1, Canada

^7^Centre for Innate Immunity and Infectious Diseases, Hudson Institute of Medical Research, Clayton, Victoria, Australia

^8^Department of Molecular and Translational Science, Monash University, Clayton, Victoria, Australia

*These authors contributed equally

**Whole cell lysis of bacterial samples.** Bacteria were grown overnight on LB plates. Plates were flooded with 5 ml of pre-chilled sterile phosphate-buffered saline (PBS) and colonies removed with a cell scraper. Cells were washed 3 times in PBS and collected by centrifugation at 10,000 x g at 4˚C then snap frozen. Frozen whole cell samples were resuspended in 4% SDS, 100 mM Tris pH 8.0, 20 mM DTT and boiled at 95˚C with shaking at 2000 rpm for 10 minutes. Samples were then clarified by centrifugation at 17,000 x g for 10 minutes, the supernatant collected, and protein concentration determined by bicinchoninic acid assay (Thermo Scientific Pierce). 200 μg of protein from each sample was acetone precipitated by mixing 4 volumes of ice-cold acetone with one volume of sample. Samples were precipitated overnight at -20˚C and then centrifuged at 16,000 x g for 10 minutes at 0˚C. The precipitated protein pellets were then resuspended in 80% ice-cold acetone and precipitated at -20˚C. for an additional 4 hours. Samples were spun down at 17,000 x g for 10 minutes at 0˚C to collect precipitated protein, the supernatant discarded, and excess acetone evaporated at 65˚C for 5 minutes.

**Enrichment of DNA bound proteins.** Bacteria were grown and washed as above then tumbled for 20 minutes at room temperature in 1% formaldehyde in PBS in accordance with the protocol of Qin *et al* (1). Cross-linking was quenched with 125 mM (final concentration) glycine in PBS for 5 minutes with tumbling. Cells were collected and washed twice with ice-cold PBS and then DNA isolated using a Zymo genomic DNA clean-up kit according to the manufacturer’s instructions with the exception of the DNA elution. DNA elution and reversal of DNA-protein cross-linking was undertaken by incubating the column at 68˚C for 1 hour with protein elution buffer (2% SDS, 0.5 M β-mercaptoethanol and 300 mM Tris pH 8.5) according to Déjardin *et al* (2). Eluted proteins were collected by centrifugation and acetone precipitated by mixing 4 volumes of ice-cold acetone with one volume of sample. Samples were precipitated overnight at -20˚C and then spun down at 16,000 x g for 10 minutes at 0˚C. The precipitated protein pellets were precipitated with 80% ice-cold acetone and resuspended as indicated above.

**Peptidomic analysis of bacterial samples.** Bacterial strains were grown and washed as above then solubilized by boiling in guanidinium chloride lysis buffer (6 M GdmCl, 100 mM Tris pH 8.5, 10 mM tris(2-carboxyethyl)phosphine, 40 mM 2-chloroacetamide) according to the protocol of Humphrey *et al* (3). Peptidomic samples were isolated according to Parker *et al* (4). Briefly, guanidinium chloride lysis buffer solubilised lysates were precipitated with 20% trichloroacetic acid on ice for 3 hours, then sample were centrifuged at 10,000 x g for 10 minutes at 0˚C, the resulting supernatant collected and peptide isolated with tC18 columns (Waters). Bound peptides were eluted with 50% acetonitrile (ACN), 0.1% FA, dried and stored at -20˚C.

**Digestion of complex protein lysates.** Dried protein pellets were resuspended in 6 M urea, 2 M thiourea, 40 mM NH_4_HCO_3_ and reduced / alkylated prior to digestion with Lys-C (1/200 w/w) then trypsin (1/50 w/w) overnight as previously described (5). Digested samples were acidified to a final concentration of 0.5% formic acid and desalted with home-made high-capacity StageTips composed on 5 mg Empore™ C18 material (3M, Maplewood, Minnesota) and 5 mg of OLIGO R3 reverse phase resin (Thermo Fisher Scientific) as described (6, 7). Bound peptides were eluted with Buffer B (80% ACN, 0.1% FA), dried and stored at -20˚C.

**Reversed phase LC-MS.** Purified peptides were resuspended in Buffer A* (2% ACN, 0.1% trifluoroacetic acid) and separated using a two-column chromatography set up comprising a PepMap100 C18 20 mm x 75 μm trap and a PepMap C18 500mm x 75μm analytical column (Thermo Scientific). Samples were concentrated onto the trap column at 5 μl/minute with Buffer A (2% ACN, 0.1%FA) for 5 minutes and infused into either an Orbitrap Elite™ Mass Spectrometer (Thermo Scientific), an Orbitrap Fusion Lumos Tribrid™ Mass Spectrometer (Thermo Scientific) or an Q-exactive plus™ Mass Spectrometer (Thermo Scientific) at 300 nl/minute via the analytical column using an Dionex Ultimate 3000 UPLC (Thermo Scientific). For whole cell proteomics analysis on the Orbitrap Elite™ 210 minute gradients were run altering the buffer composition from 1% buffer B to 28% B over 180 minutes, then from 28% B to 40% B over 10 minutes, then from 40% B to 100% B over 2 minutes, the composition was held at 100% B for 3 minutes, and then dropped to 3% B over 5 minutes and held at 3% B for another 10 minutes. The Orbitrap Elite™ was operated in a data-dependent mode automatically switching between the acquisition of a single Orbitrap MS scan (60,000 resolution) followed by 5 data-dependent HCD MS-MS events (resolution 15 k AGC target of 4 x 10^5^ with a maximum injection time of 250 ms, NCE 35) with 30 seconds dynamic exclusion enabled. For whole cell proteomics analysis on the Fusion™ 120 minute gradients were run altering the buffer composition from 1% buffer B to 28% B over 90 minutes, then from 28% B to 40% B over 10 minutes, then from 40% B to 100% B over 2 minutes, the composition was held at 100% B for 3 minutes, and then dropped to 3% B over 5 minutes and held at 3% B for another 10 minutes. The Fusion™ was operated in a data-dependent acquisition switching between the acquisition of an Orbitrap MS scan (120,000 resolution) every 3 seconds and HCD undertaken for each selected precursor (maximum fill time 100 ms, AGC 5 x 10^4^ with a resolution of 15,000 for Orbitrap MS-MS scans) with 30 seconds dynamic exclusion enabled.

DNA bound proteomic analysis was undertaken on both a Orbitrap Elite™ (for data-dependent acquisition experiments) and Q-exactive plus™ (for data-independent acquisition experiments) with 90 minute gradients run altering the buffer composition from 1% buffer B to 28% B over 60 minutes, then from 28% B to 40% B over 10 minutes, then from 40% B to 100% B over 2 minutes, the composition was held at 100% B for 3 minutes, and then dropped to 3% B over 5 minutes and held at 3% B for another 10 minutes. For data-dependent acquisition experiments the Elite™ Mass Spectrometer was operated in a data-dependent mode automatically switching between the acquisition of a single Orbitrap MS scan (60,000 resolution) followed by 10 data-dependent CID MS-MS events (analysed in the ITMS, maximum injection time of 100 ms, NCE 35). To enable the robust quantification of CepR data-independent acquisition was undertaken using parallel reaction monitoring (PRM (8)) monitoring tryptic peptides of BCAL3530 (DNA-binding protein HU-alpha), BCAL0462 (Putative DNA topoisomerase III), BCAM0904 (DNA polymerase I), BCAM1868 (CepR) and BCAM1870 (CepI), see PRM list below. BCAL3530, BCAL0462, BCAM0904 were included as positive controls to ensure equal DNA enrichment while CepI is a non-DNA binding control. Data-independent acquisition was performed by switching between the acquisition of a single Orbitrap MS scan (70,000 resolution, m/z 350-1400) and HCD MS/MS events of each PRM precursor (maximum fill time 110ms, AGC 2 x 10^5^ with a resolution of 35,000 for Orbitrap MS-MS scans).

| **m/z** | **Charge** | **Peptide sequence** | **Protein accessions (Protein names)** |
| --- | --- | --- | --- |
| 815.9485 | 2 | AQTGETLDTLLEVIK | BCAL3530 (HupA) |
| 770.3937 | 2 | GDAVQLIGFGSFGSGK | BCAL3530 (HupA) |
| 751.3964 | 2 | QELIDAVAAQTGASK | BCAL3530 (HupA) |
| 551.3174 | 3 | TDEIAAVALVTKPPAR | BCAL0462 |
| 671.3904 | 2 | VQTPTLSIVVER | BCAL0462 |
| 399.2 | 3 | ESHNYLPHAK | BCAL0462 |
| 499.2825 | 2 | AAYLPVAHR | BCAM0904 (PolA) |
| 1072.5779 | 2 | IIDYLALIGDTVDNVPGVEK | BCAM0904 (PolA) |
| 401.2163 | 2 | FGVPPER | BCAM0904 (PolA) |
| 1090.0445 | 2 | VFVEQLGWALPSANESFER | BCAM1870 (CepI) |
| 657.91 | 2 | LLPTTRPYLLK | BCAM1870 (CepI) |
| 397.88 | 3 | LPHELAADLGR | BCAM1870 (CepI) |
| 543.26 | 2 | DDTVYVFAR | BCAM1870 (CepI) |
| 825.915 | 2 | DFGLSVGVAQSSWAAR | BCAM1868 (CepR) |
| 476.59 | 3 | TVNFHVNNILEK | BCAM1868 (CepR) |
| 578.83 | 2 | LSPAAGVTLTAR | BCAM1868 (CepR) |
| 502.79 | 2 | GAFGLLSIAR | BCAM1868 (CepR) |

**PRM peptide list:** The peptides selectively monitored by PRM are provided with the m/z, charge, amino acid sequence and protein

Peptidomic analysis was undertaken on an Orbitrap Elite™ with 120 minute gradients run altering the buffer composition from 1% buffer B to 28% B over 90 minutes, then from 28% B to 40% B over 10 minutes, then from 40% B to 100% B over 2 minutes, the composition was held at 100% B for 3 minutes, and then dropped to 3% B over 5 minutes and held at 3% B for another 10 minutes. The Elite™ Mass Spectrometer was operated in a data-dependent mode automatically switching between the acquisition of a single Orbitrap MS scan (60,000 resolution) followed by 5 data-dependent CID and HCD MS-MS events (both acquired in the Orbitrap for each precursor at a resolution of 15 k AGC target of 4 x 10^5^ with a maximum injection time of 250 ms, NCE 35) with 30 seconds dynamic exclusion enabled.

**Data analysis***:* MS datasets were processed using MaxQuant (v1.5.5.1 or 1.5.3.30 (9)). Database searching was carried out against the reference *B. cenocepacia* strain J2315 (<https://www.uniprot.org/proteomes/UP000001035>*,* downloaded September 25^th^ 2017) and the K56-2Valvano proteome (10) (http://www.uniprot.org/taxonomy/985076, downloaded from NCBI February 15^th^ 2013). All tryptic digest searchers were undertaken using “Trypsin” enzyme specificity, carbamidomethylation of cysteine as a fixed modification; oxidation of methionine, acetylation of protein N-terminal trypsin/P cleavage with a maximum of 2 missed cleavages. Peptidomic analysis was undertaken using “Unspecific” enzyme specificity and allowing the presence of *O*-linked glycosylation BC glycan 1 (elemental composition: C_22_O_15_H_36_N_2_, mass: 568.2115) and BC glycan 2 (elemental composition: C_26_O_18_H_40_N_2_, mass: 668.2276) at serine residues. To enhance the identification of peptides between samples, the Match between Runs option was enabled with a precursor match window set to 2 minutes and an alignment window of 10 minutes. For label free quantitation the MaxLFQ option in Maxquant (11) was enabled in addition to the re-quantification module. The resulting outputs were processed in the Perseus (v1.5.0.9) (12) analysis environment to remove reverse matches and common protein contaminates prior to further analysis. For label-free based quantitative (LFQ) comparisons missing values were imputed using Perseus and data z-scored to enabled visualization in heat maps using R (<https://www.r-project.org/)>. Pearson correlations were performed on non-imputed dataset with clustering and visualization performed in Perseus. Clustering of Pearson correlations data was undertaken using the Euclidean distance and complete linkage with pre-processing with k-means in Perseus. Enrichment analysis was undertaken using Fisher exact test in Perseus with Gene Ontology (GO) terms, gene names, subcellular location [CC], signal peptide status, lipidation status, intramembrane status and keywords associated with each protein obtain from uniport (*B. cenocepacia* strain J2315 proteomes: UP000001035, downloaded September 25^th^ 2017). Virulence associated genes were compiled from genes defined as Virulence associated in the *Burkholderia* Genome Database (13) (J2315, Downloaded 3*^rd^* October 2017) and the genome analysis of *B. cenocepacia* strain J2315 (14) (Supplementary Table 1). CepR regulated proteins were defined as those proteins which were previously reported by O’Grady *et al* as differential regulated in K56-2 Δ*cepR* at stationary phase (15). Proteomics data sets have been deposited to the ProteomeXchange Consortium via the PRIDE (16) partner repository with the dataset identifier PXD014429, PXD014516, PXD014581, PXD014614 and PXD014700. For a complete description of each PRIDE dataset see Table 4.

**References**

1. Qin H, Wang Y. 2009. Exploring DNA-binding proteins with in vivo chemical cross-linking and mass spectrometry. J Proteome Res 8:1983-91.

2. Dejardin J, Kingston RE. 2009. Purification of proteins associated with specific genomic Loci. Cell 136:175-86.

3. Humphrey SJ, Azimifar SB, Mann M. 2015. High-throughput phosphoproteomics reveals in vivo insulin signaling dynamics. Nat Biotechnol 33:990-5.

4. Parker BL, Burchfield JG, Clayton D, Geddes TA, Payne RJ, Kiens B, Wojtaszewski JFP, Richter EA, James DE. 2017. Multiplexed Temporal Quantification of the Exercise-regulated Plasma Peptidome. Mol Cell Proteomics 16:2055-2068.

5. Scott NE, Parker BL, Connolly AM, Paulech J, Edwards AV, Crossett B, Falconer L, Kolarich D, Djordjevic SP, Hojrup P, Packer NH, Larsen MR, Cordwell SJ. 2011. Simultaneous Glycan-Peptide Characterization Using Hydrophilic Interaction Chromatography and Parallel Fragmentation by CID, Higher Energy Collisional Dissociation, and Electron Transfer Dissociation MS Applied to the N-Linked Glycoproteome of Campylobacter jejuni. Mol Cell Proteomics 10:M000031MCP201.

6. Ishihama Y, Rappsilber J, Mann M. 2006. Modular stop and go extraction tips with stacked disks for parallel and multidimensional Peptide fractionation in proteomics. J Proteome Res 5:988-94.

7. Rappsilber J, Mann M, Ishihama Y. 2007. Protocol for micro-purification, enrichment, pre-fractionation and storage of peptides for proteomics using StageTips. Nat Protoc 2:1896-906.

8. Peterson AC, Russell JD, Bailey DJ, Westphall MS, Coon JJ. 2012. Parallel reaction monitoring for high resolution and high mass accuracy quantitative, targeted proteomics. Mol Cell Proteomics 11:1475-88.

9. Cox J, Mann M. 2008. MaxQuant enables high peptide identification rates, individualized p.p.b.-range mass accuracies and proteome-wide protein quantification. Nat Biotechnol 26:1367-72.

10. Varga JJ, Losada L, Zelazny AM, Kim M, McCorrison J, Brinkac L, Sampaio EP, Greenberg DE, Singh I, Heiner C, Ashby M, Nierman WC, Holland SM, Goldberg JB. 2013. Draft Genome Sequences of Burkholderia cenocepacia ET12 Lineage Strains K56-2 and BC7. Genome Announc 1.

11. Cox J, Hein MY, Luber CA, Paron I, Nagaraj N, Mann M. 2014. Accurate proteome-wide label-free quantification by delayed normalization and maximal peptide ratio extraction, termed MaxLFQ. Mol Cell Proteomics 13:2513-26.

12. Tyanova S, Temu T, Sinitcyn P, Carlson A, Hein MY, Geiger T, Mann M, Cox J. 2016. The Perseus computational platform for comprehensive analysis of (prote)omics data. Nat Methods 13:731-40.

13. Winsor GL, Khaira B, Van Rossum T, Lo R, Whiteside MD, Brinkman FS. 2008. The Burkholderia Genome Database: facilitating flexible queries and comparative analyses. Bioinformatics 24:2803-4.

14. Holden MT, Seth-Smith HM, Crossman LC, Sebaihia M, Bentley SD, Cerdeno-Tarraga AM, Thomson NR, Bason N, Quail MA, Sharp S, Cherevach I, Churcher C, Goodhead I, Hauser H, Holroyd N, Mungall K, Scott P, Walker D, White B, Rose H, Iversen P, Mil-Homens D, Rocha EP, Fialho AM, Baldwin A, Dowson C, Barrell BG, Govan JR, Vandamme P, Hart CA, Mahenthiralingam E, Parkhill J. 2009. The genome of Burkholderia cenocepacia J2315, an epidemic pathogen of cystic fibrosis patients. J Bacteriol 191:261-77.

15. O'Grady EP, Viteri DF, Malott RJ, Sokol PA. 2009. Reciprocal regulation by the CepIR and CciIR quorum sensing systems in Burkholderia cenocepacia. BMC Genomics 10:441.

16. Vizcaino JA, Csordas A, del-Toro N, Dianes JA, Griss J, Lavidas I, Mayer G, Perez-Riverol Y, Reisinger F, Ternent T, Xu QW, Wang R, Hermjakob H. 2016. 2016 update of the PRIDE database and its related tools. Nucleic Acids Res 44:D447-56.
